# The antitumor mechanisms of glabridin and drug delivery strategies for enhancing its bioavailability

**DOI:** 10.3389/fonc.2024.1506588

**Published:** 2024-12-11

**Authors:** Chong Li, Yu Wang, Wenjing Zhang, Xiaoman Yang, Yufang Wang, Guanqun Hou, Dongli Wang, Bingbing Han, Yimin Zhang

**Affiliations:** ^1^ College of Traditional Chinese Medicine, Shandong University of Traditional Chinese Medicine, Jinan, Shandong, China; ^2^ Department of Spleen and Stomach, Hospital Affiliated to Shandong University of Traditional Chinese Medicine, Jinan, China; ^3^ Key Laboratory of Traditional Chinese Medicine Classical Theory, Ministry of Education, Shandong University of Traditional Chinese Medicine, Jinan, China

**Keywords:** glabridin, antitumor, novel delivery system, mechanism, safety evaluation

## Abstract

Glabridin, a flavonoid derived from the plant *Glycyrrhiza glabra*, has garnered significant attention due to its diverse pharmacological effects, including antioxidant, antibacterial, anti-inflammatory, hypolipidemic, and hypoglycemic activities. Studies have shown that glabridin exhibits substantial antitumor activity by modulating the proliferation, apoptosis, metastasis, and invasion of cancer cells through the targeting of various signaling pathways, thus indicating its potential as a therapeutic agent for malignant tumors. To enhance its solubility, stability, and bioavailability, several drug delivery systems have been developed, including liposomes, cyclodextrin inclusion complexes, nanoparticles, and polymeric micelles. These de.livery systems have shown promise in preclinical studies but face challenges in clinical translation, such as issues with biocompatibility, delivery efficiency, and long-term stability. A comprehensive analysis of the antitumor mechanism of glabridin and its novel drug delivery system is still lacking. Therefore, the authors performed a comprehensive review of recent literature on the antitumor effects of glabridin and its novel drug delivery systems, covering the antitumor mechanism, action targets, and novel drug delivery systems, offering new theoretical insights and development directions for its further advancement and clinical application.

## 1 Introduction

The incidence of malignant tumors is increasing worldwide due to aging populations, environmental degradation, and lifestyle changes. There were close to 20 million new cases of cancer in the year 2022 alongside 9.7 million deaths from cancer. The estimates suggest that approximately one in five men or women develop cancer in a lifetime, whereas around one in nine men and one in 12 women die from it (1). Malignant tumors pose a severe threat to physical and mental health, as well as life safety, and are a leading cause of death (2). Surgery, radiotherapy, chemotherapy, immunotherapy, and gene-targeted therapies are commonly used to treat malignant tumors (3). Despite improvements in survival rates with these treatments, challenges such as recurrence, radiotherapy-related toxicity, and drug resistance remain. Therefore, identifying new, effective antitumor drugs and exploring their mechanisms is crucial for progress.

In recent years, Traditional Chinese Medicine (TCM) has gained recognition for its role in cancer treatment, with several anti-tumor compounds identified in traditional remedies (4–6). This underscores the substantial potential of TCM for anti-cancer development, warranting further investigation. Glabridin, a flavonoid extracted from the medicinal plant Glycyrrhiza glabra, pharmacological studies have demonstrated that, beyond its antioxidant (7), anti-inflammatory (8), osteoprotective (9), hypoglycemic (10), and neuroprotective effects (11, 12), glabridin also exhibits notable antitumor activity (13).

Compared with other anti-tumor compounds derived from Chinese herbal medicine, glabridin shows significant advantages. First, glabridin has a broad spectrum of pharmacological effects and is widely used in the food (14) and cosmetics (15) industries, in addition to the medical field, demonstrating considerable development potential. Second, from a pharmacokinetic perspective, some common anti-tumor compounds derived from traditional Chinese medicine, such as curcumin (16), baicalin (17), and quercetin (18), have poor water solubility, low absorption, low bioavailability, rapid metabolism, and high systemic clearance, which limits their therapeutic efficacy. Research has shown that glabridin undergoes primary metabolism via glucuronidation (19), with intestinal microsomes exhibiting only 1/15 to 1/20 of the metabolic capacity of liver microsomes, and circulates in the bloodstream primarily as glycoside conjugates, indicating a higher bioavailability compared to other flavonoids, such as quercetin (20). In addition, glabridin is broadly distributed across various tissues and is detectable in all tissues except the brain (21), suggesting its potential for therapeutic effects across multiple tissues. Studies have shown that glabridin’s binding affinity for the human estrogen receptor is comparable to genistein’s (the best-known phytoestrogen) (22) and that abnormally high estrogen levels are associated with an increased incidence of certain cancers (23), particularly breast cancer (24) and endometrial cancer (25). This suggests that glabridin has significant potential as a therapeutic agent for breast and endometrial cancer. In summary, the distinct characteristics and therapeutic advantages of glabridin suggest promising applications in anti-tumor therapies.

**Figure 1:**
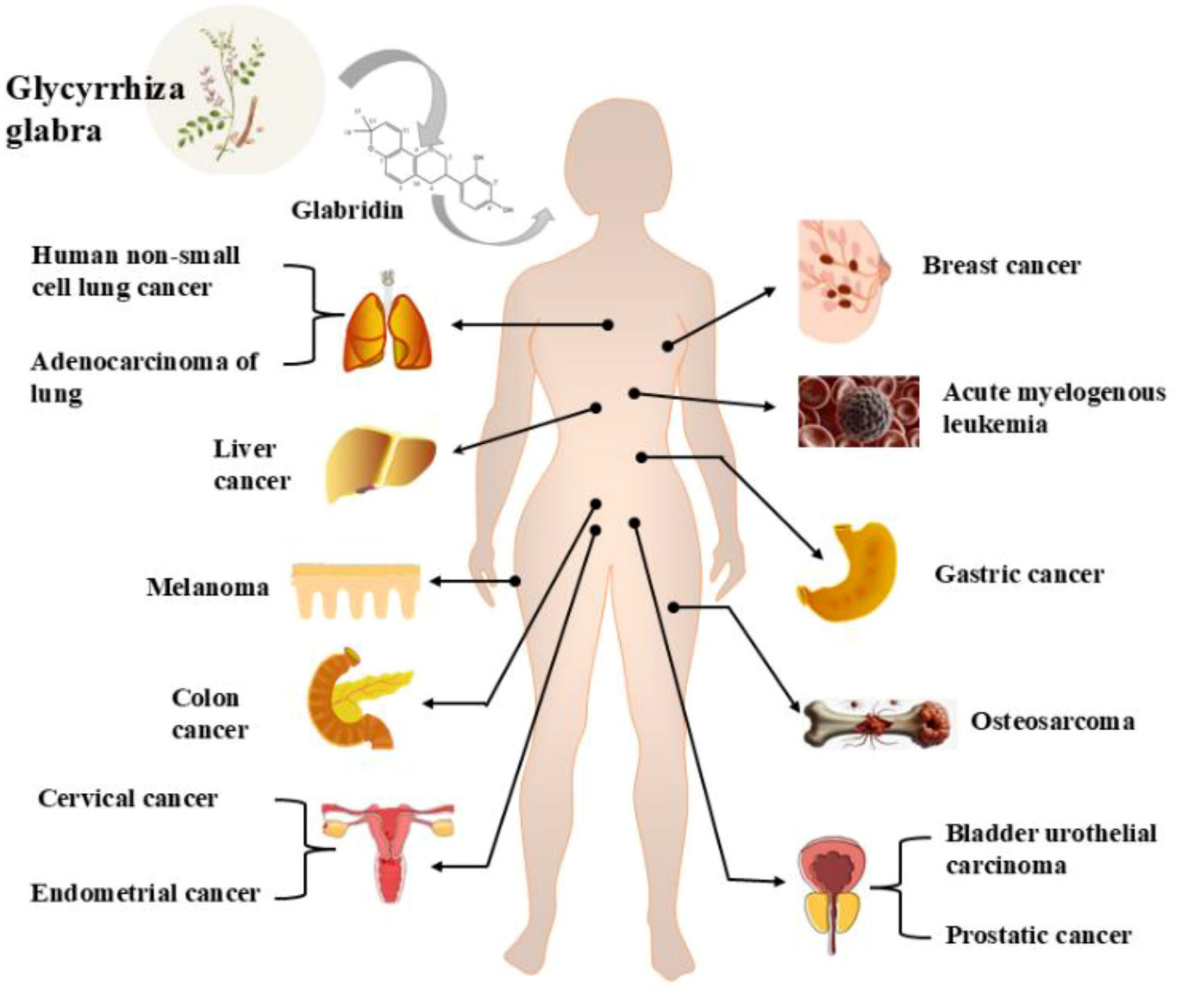
Glabridin treats different cancers.

In recent years, several reviews have examined the pharmacological effects of glabridin and its derivatives (22, 26), with a primary focus on the common pharmacological mechanisms of glabridin’s action. However, reviews specifically addressing the unique molecular targets of glabridin in various cancer types and its integration with novel drug delivery systems are still lacking. Therefore, this review aims to examine the anticancer mechanisms, molecular targets, and novel drug delivery systems of glabridin, providing the latest advancements and comprehensive insights in the field of anti-malignant tumors and drug delivery, with the goal of offering a scientific basis for its further development and clinical application. For information on the types of cancers treated by glabridin, please refer to **Figure 1**.

## 2 Chemical properties of glabridin

**Figure 2:**
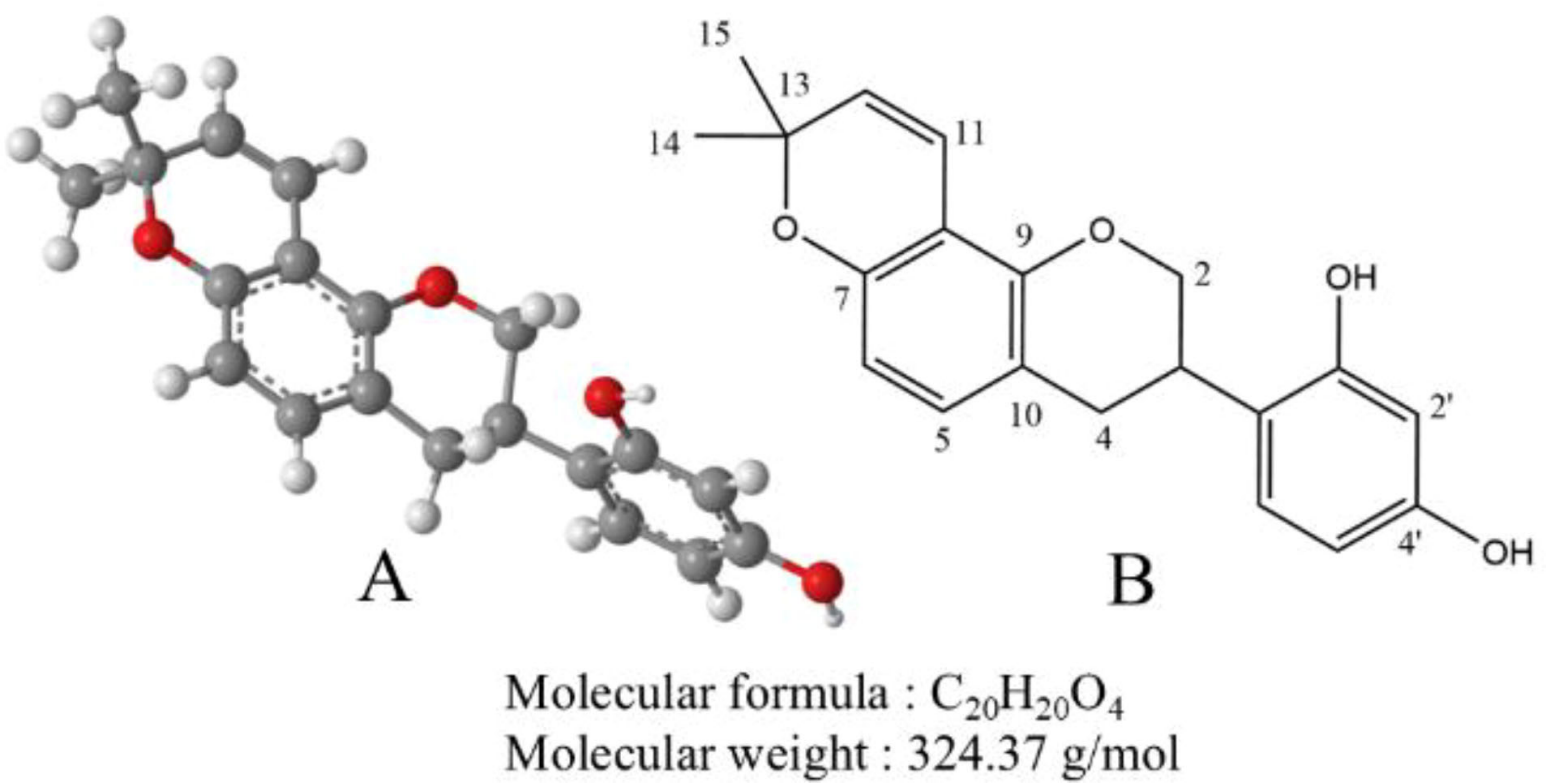
**(A)** The ball-and-stick model of the molecular structure of glabridin. The gray, red, and white balls represent carbon, oxygen, and hydrogen atoms, respectively. **(B)** The chemical structure of glabridin.

Glabridin is a flavonoid primarily extracted from the root of *Glycyrrhiza glabra*, commonly known as licorice. It has a molecular formula of C_20_H_20_O_4_ and a molecular weight of 324.37 g/mol. Glabridin appears as a pale yellow powder, insoluble in water but soluble in organic solvents such as ethanol, methanol, and acetone (27). Its structure features a flavone backbone with hydroxyl groups at the 7 and 4’ positions. The chemical structure of glabridin underpins its biological activities, including antioxidant and antitumor properties. Light exposure is the primary factor affecting the stability of glabridin. Additionally, interactions between temperature and pH, temperature and humidity, and light exposure and pH can further accelerate the degradation of glabridin (28). The chemical formula of glabridin is shown in **Figure 2**.

## 3 Pharmacokinetics of glabridin

Glabridin has poor water solubility, and low bioavailability, and is prone to isomerization under light, temperature, and humidity. Its pharmacological mechanism is not yet fully understood. Pharmacokinetic analysis of human oral metabolism experiments indicated that glabridin reached a maximum concentration of approximately 4 hours post-administration in blood, with a half-life (T1/2) of about 10 hours (29). *In vivo* studies show glabridin peaks at 87 nmol/L 1-hour post-administration, with a half-life (T1/2) of 8.2 hours and bioavailability (AUCinf) of 0.825 μM·h (20). Animal experiments have shown that glabridin has an oral bioavailability of 6.63% and a first-pass effect in the liver of 62.12% (21). Additionally, studies have identified several metabolic pathways for glabridin, including glucuronidation, demethylation, hydroxylation, and sulfation. Glabridin metabolites have been detected in various biological fluids and tissues, including plasma from the abdominal aorta and hepatic portal vein, as well as in the brain, liver, heart, lung, spleen, kidney, bile, urine, and feces (30). The high first-pass effect in the liver and extensive metabolism may account for the low oral bioavailability of glabridin. Therefore, enhancing the bioavailability of glabridin remains a pressing challenge.

## 4 Safety evaluation of glabridin

As research progresses, the safety of glabridin has increasingly become a focal point of investigation. There are no *in vivo* toxicity data on glabridin, but safety assessments exist for Licorice flavonoid oil (LFO), where glabridin is a major marker compound. LFO is marketed as Glavonoid and approved by the European Food Safety Authority as a novel food ingredient (13).

Fumiki Aoki et al. evaluated the clinical safety of LFO. Hematological and related biochemical indicators showed that LFO doses up to 1200 mg/day were safe and well tolerated over a 4-week period. Subsequent pharmacokinetic studies showed that LFO exhibited linear pharmacokinetics across doses ranging from 300 to 1200 mg per person (29).

Animal studies also demonstrate that LFO is highly safe. In an 8-week study involving LFO intervention in obese mice, there were no significant differences in the liver, kidney, and spleen weights between the treated and control groups, suggesting that glabridin is non-toxic (31).

Nakagawa et al. (32) conducted a 90-day oral toxicity study in rats using an LFO concentrate containing 2.9% glabridin. The results showed that the no-observed-adverse-effect level (NOAEL) was 800 mg/kg/day for female rats and 400 mg/kg/day for male rats. In addition, Nakagawa et al. (33) used three different methods to study the genotoxicity of LFO in another experiment. In the bacterial reverse mutation assay, using four strains of *Salmonella typhimurium* and *Escherichia coli*, LFO did not increase the number of colonies in any of the test strains. In the Chinese hamster lung (CHL/IU) cell chromosome aberration assay, only LFO at concentrations exceeding 0.6 mg/mL induced structural chromosomal aberrations in the short-term test, while LFO at lower concentrations did not cause chromosomal aberrations in mice. In the bone marrow micronucleus assay conducted on male F344 rats, doses up to 5000 mg/kg/day did not significantly increase the frequency of micronucleated polychromatic erythrocytes (MNPCE). Therefore, LFO demonstrates good tolerance and safety, with a low incidence of adverse reactions, making it suitable for long-term disease treatment.

It is important to note that, while glabridin is the primary marker compound of LFO, the current safety evaluation studies of LFO do not fully establish the safety of glabridin. Therefore, further animal studies are needed to clarify the metabolic pathway of glabridin and evaluate its safety *in vivo*. In preclinical studies, glabridin demonstrated good tolerability within a specific dose range, with no significant weight loss or organ toxicity. In a mouse model, doses of 10-20 mg/kg did not produce significant toxic effects and remained unchanged in liver and kidney function or hematological indicators (34–38). However, the potential chronic toxicity of glabridin with long-term use has not been fully assessed. *In vitro* experiments have shown that glabridin has significant inhibitory effects on cancer cells at concentrations of 10-100 μM and effectively induces apoptosis. Additionally, the IC50 values of glabridin vary across different cancer cell lines, indicating that its inhibitory effect is dose- and cell-type dependent. Future studies should further assess the long-term toxicity of glabridin, determine its maximum tolerated dose, and establish its safety margin through additional *in vitro* and *in vivo* experiments. Furthermore, potential interactions between glabridin and other drugs need to be thoroughly evaluated at the preclinical stage to prevent increased toxicity from drug interactions in combination therapies.

## 5 Glabridin anti-tumor mechanisms

### 5.1 Inhibition of tumor cell invasion and metastasis

**Figure 3:**
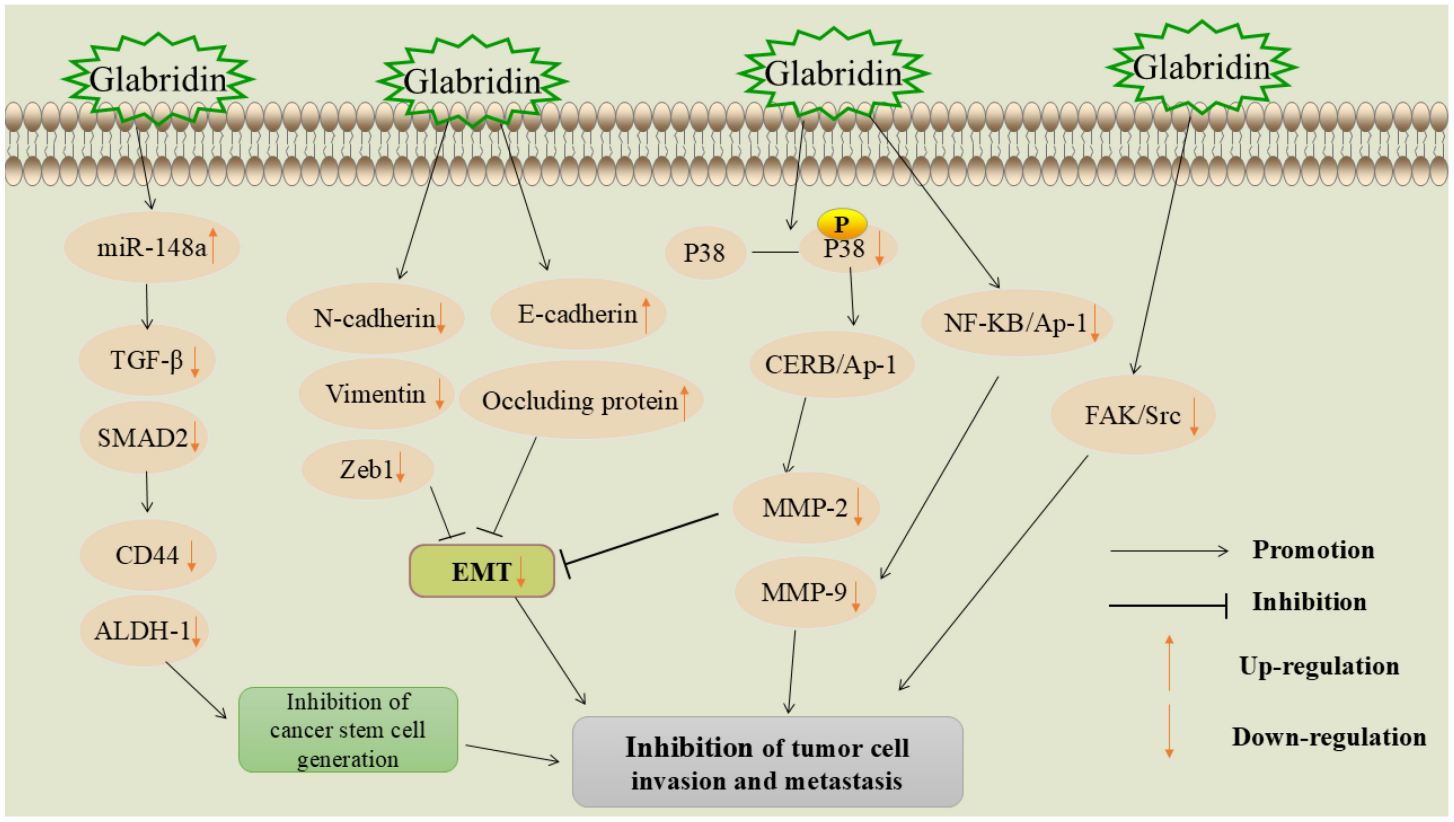
Mechanism of glabridin inhibition of tumor cell metastasis and invasion.

Invasion and metastasis refer to the spread of tumor cells from the primary site to distant tissues or organs via lymph or blood circulation and are the leading causes of cancer-related deaths (39). The first-line treatment for metastatic tumors is systemic chemotherapy, which often causes severe side effects, such as organ failure and high infection rates (40). Therefore, discovering new methods to inhibit the invasion and metastasis of malignant tumor cells is of paramount importance in the fight against cancer. The mechanism of glabridin inhibition of tumor cell metastasis and invasion is shown in **Figure 3**.

#### 5.1.1 Promotion of microRNA-148a signaling pathway

MicroRNAs (miRNAs) are small non-coding RNAs that regulate gene expression and serve as cancer biomarkers. Studies show that miR-148a suppression is linked to metastasis in various tumors and upregulation of metastasis-related genes (41). Transforming growth factor-β (TGF-β) plays an important role in tissue and organ development, as well as cell proliferation, differentiation, and apoptosis. It can affect epithelial and endothelial cell differentiation, increase immune cell recruitment, and is an important cytokine involved in the activation of cancerous mesenchyme (42). Small mothers against decapentaplegic (SMAD) proteins are key proteins that conduct TGF-β signaling from cell surface receptors to the nucleus (43).

Jiang et al. found that glabridin significantly inhibited the adhesion-dependent growth and sphere formation of HepG2 and MHCC97H hepatocellular carcinoma cells (44). It increased miR-148a expression in a dose- and time-dependent manner, targeting TGF-β and SMAD protein activation, and reducing tumor cell invasion and metastasis. Another study showed that glabridin reduced miR-148a methylation and upregulated its expression in breast cancer cells, inhibiting the TGF-β/SMAD pathway and suppressing metastasis (34).

miR-148a significantly inhibits tumor cell invasion and metastasis by targeting the TGF-β/SMAD signaling pathway. However, the mechanisms of action of miR-148a across different cancer types have not been fully elucidated. miR-148a not only exerts its effects within cancer cells, but may also influence immune escape mechanisms by modulating immune responses in the tumor microenvironment (45). Whether glabridin can alter the tumor microenvironment by regulating miR-148a and thereby provide a new target for immunotherapy requires further investigation.

#### 5.1.2 Blocking the epithelial-mesenchymal transition process

EMT is a crucial process involving the transformation of epithelial cells, which alters intercellular adhesion patterns and affects cell proliferation and differentiation. EMT is closely associated with embryonic development, tissue repair, and cancer progression (46). Aberrant activation of EMT is linked to malignant properties of tumor cells during cancer progression and metastasis, such as increased tumor cell migration and invasiveness, enhanced tumor stemness, and greater resistance to chemotherapy and immunotherapy (47). Therefore, targeting and blocking the EMT process is crucial for controlling tumor invasion and metastasis.

Glabridin inhibited the proliferation, migration, and invasion of lung adenocarcinoma cells A549 (35). Further studies revealed that glabridin decreased the expression of neural cadherin (N-cadherin) and vimentin, and upregulated the expression of epithelial cadherin (E-cadherin) in tumor cells. N-cadherin and vimentin are positively correlated with EMT and are considered its markers (48, 49), whereas E-cadherin acts as a tumor suppressor protein (50). Another study found that glabridin inhibited EMT in breast cancer cells by upregulating E-cadherin and occludin, while downregulating vimentin, E-box-binding zinc finger protein 1 (Zeb1), and other critical EMT markers, thereby exerting a controlling effect on tumor invasion and metastasis (36).

#### 5.1.3 Inhibition of focal adhesion kinase/sarcoma receptor coactivator signaling pathway

FAK is a key signaling molecule involved in cell adhesion, migration, proliferation, and survival. Its overactivation in various cancers makes it an important target for developing new anti-cancer therapies (51). Src is a non-receptor tyrosine kinase that phosphorylates tyrosine residues on proteins. Unlike receptor tyrosine kinases, Src is not membrane-bound and acts at various intracellular sites, affecting many cellular processes. Src is often overactive in cancers, leading to uncontrolled cell proliferation, increased invasion and metastasis, and resistance to apoptosis (52). FAK typically forms a complex with Src, jointly regulating cell growth, survival, and motility. Aberrant activation of FAK/Src signaling is frequently observed in aggressive tumors (53).

Glabridin was found to inhibit the invasion and metastasis of human non-small cell lung cancer A549 cells (54). Studies have shown that glabridin inhibits lung cancer cell invasion and metastasis by reducing FAK and Src activities, leading to FAK/Src complex inactivation. Additionally, glabridin can suppress the invasion, metastasis, and angiogenesis of breast cancer cells through the FAK/Src pathway (55), the specific mechanism is that glabridin inhibits the migration and invasion of cancer cells by reducing the phosphorylation of key proteins. It also regulates cytoskeleton reorganization to prevent cell movement by inhibiting the Ras homolog family member A(RhoA)/Rho-associated coiled-coil containing protein kinase (ROCK) signaling pathway and reducing the phosphorylation of myosin light chain (MLC). In addition, glabridin directly inhibits the migration and tube formation of vascular endothelial cells, thereby effectively inhibiting angiogenesis. These multi-pathway effects suggest that glabridin has a strong potential to inhibit breast cancer invasion and metastasis.

The FAK/Src signaling pathway plays a crucial role in tumor cell migration, invasion, and metastasis. Glabridin exerts anti-metastatic effects in various tumors by inhibiting the activation of the FAK/Src complex. Since FAK/Src also plays a key role in tumor angiogenesis (56), future research should focus on the inhibition of tumor angiogenesis by glabridin through its effect on FAK/Src, to assess its novel potential as a therapeutic strategy.

#### 5.1.4 Inhibition of matrix metalloproteinases family proteases

MMPs have been shown to play an important role in the abnormal proliferation, local invasion, and metastasis of tumor cells (57). They can degrade almost all protein components in the extracellular matrix (ECM), destroy the histological barrier to tumor cell invasion, and act as proteases that remodel the extracellular matrix, driving the cell invasion process (58).

Jie et al. (59) found that glabridin inhibits the invasion and migration of osteosarcoma cells. The specific mechanism involves down-regulating p38 mitogen-activated protein kinase (p38 MAPK) and c-Jun N-terminal kinase (JNK) phosphorylation, which affects the binding of cyclic adenosine monophosphate response element-binding protein-associated protein 1 (CREB-AP1) to the promoters of matrix metalloproteinase-2 (MMP-2) and matrix metalloproteinase-9 (MMP-9), thereby inhibiting tumor cell migration and invasion. Another study found that glabridin inhibits melanoma cell proliferation and migration by reducing MMP-2 activity and expression (60). In a study involving glabridin intervention in liver cancer cells Huh7 and Sk-Hep-1, the relationship between glabridin’s regulation of MMPs and the inhibition of tumor cell invasion and migration was further verified (61). The results showed that glabridin downregulated MMP-9 by interfering with nuclear factor-κB (NF-κB) and AP-1 binding activity, ultimately inhibiting tumor cell invasion and metastasis. For detailed information, please refer to **Table 1**.

**Table 1:** Mechanism of glabridin inhibition of tumor cell invasion and metastasis.

Cancers	Effective concentrations	Cell type/Animal model	Possible mechanisms	Reference
Liver cancer	10, 20 μM	Hepatoma carcinoma cell lines: HepG2, MHCC97H	Glabridin inhibits the TGF-β/SMAD2 pathway by upregulating miR-148a, reducing cancer stem cell markers (CD44, EpCAM), and impairing self-renewal, tumor sphere formation, and independent growth.	(44)
Breast cancer	20 mg/kg	BALB/c nude mice	Glabridin blocks the TGF-β/SMAD2 pathway by upregulating miR-148a, reducing the proliferation and transformation traits of cancer stem cells (CSCs). It increases epithelial markers like E-cadherin and ZO-1, inhibits EMT, and weakens cell migration and invasion. Additionally, it reduces CSC markers like CD44 and ALDH-1, inhibiting tumor self-renewal, sphere formation, and anchorage-independent growth.	(34)
	10 μM	Human breast cancer cell lines MDA-MB-231 and Hs-578T		
Adenocarcinoma of lung	12.5, 25, 50 mg/kg	BALB/c nude mice	Glabridin inhibits EMT by upregulating E-cadherin and downregulating EMT-related proteins like N-cadherin, Snail, and vimentin, reducing cancer cell migration and invasion.	(35)
	20, 40, 60, 80, 100 µM	Adenocarcinoma of lung cell: A459		
Breast cancer	10 mg/kg	Balb/C mice	Glabridin inhibits EMT by upregulating epithelial markers (E-cadherin, occludin) and downregulating mesenchymal markers (vimentin, Zeb1), effectively reducing tumor metastasis.	(36)
	10, 20, 40 μM	Breast carcinoma cell lines:4t1		
Lung Cancer	1, 2.5, 5, 10 μM	Adenocarcinoma of lung cells: A459 cells: A549, type II alveolar epithelial cells	Glabridin inhibits FAK and Src activation, blocks the FAK/Src complex, and suppresses the AKT and RhoA pathways, reducing cancer cell migration and invasion. It also limits tumor angiogenesis by inhibiting vascular endothelial cell tube formation.	(54)
Breast cancer	1, 2.5, 5, 11 μM	Human breast cancer cell lines: MDA-MB-231	Glabridin inhibits cancer cell migration and invasion by suppressing FAK and Src activation. It also reduces cell migration by decreasing RhoA and MLC activity, preventing cytoskeletal reorganization.	(55)
Osteosarcoma	5, 10, 20 μM	Human osteosarcoma cell lines: MG63	Glabridin inhibits osteosarcoma cell migration and invasion by suppressing p38 and JNK pathway activation, preventing CREB-AP1 complex formation, and reducing MMP-2 and MMP-9 expression.	(59)
Melanoma	20, 40, 60, 80, 120 μM	Mouse melanoma cell: B16F1	Glabridin reduces MMP-2 secretion, limiting tumor cell degradation of the ECM.	(60)
Liver cancer	10, 20, 40 μM	Human hepatoma cell lines: Huh7, Sk-Hep-1	Glabridin inhibits tumor cell migration and invasion by suppressing the NF-κB, AP-1, and JNK1/2 pathways, downregulating MMP-9.	(61)

### 5.2 Induction of tumor cell apoptosis

**Figure 4:**
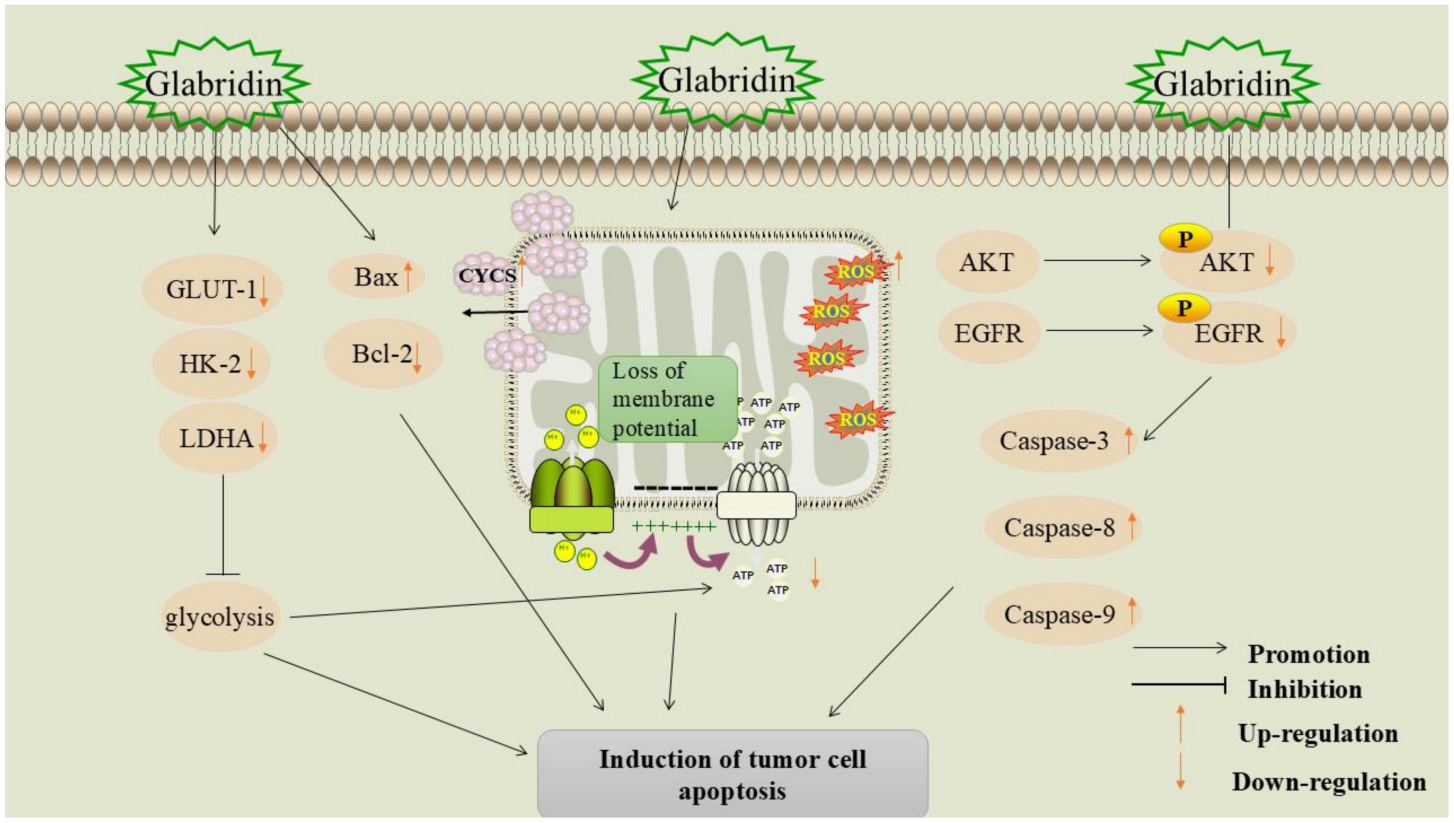
Mechanism of glabridin-induced apoptosis in tumor cells.

Apoptosis is a mechanism that maintains tissue homeostasis by removing damaged cells through programmed cell death (62). Dysregulation of the apoptosis mechanism is a significant hallmark of cancer. Apoptosis affects tumor initiation, progression, and the development of drug resistance during cancer treatment. Therefore, inducing apoptosis is a crucial component in anti-tumor strategies (63). The mechanism of glabridin-induced tumor cell apoptosis is shown in **Figure 4**.

#### 5.2.1 Inhibition of glycolysis

Energy metabolism disorders are regarded as one of the hallmarks of cancer (64). In tumor tissues, glycolysis is the main metabolic pathway for adenosine triphosphate (ATP) production. Cancer cells depend on metabolic reprogramming and transition to a “glycolytic” metabolic phenotype to sustain the energy necessary for proliferation (65, 66). Hexokinase-2 (HK-2) is the primary rate-limiting enzyme in glycolysis and a key regulator in the glucose metabolic pathway. HK-2 catalyzes the phosphorylation of glucose to produce glucose-6-phosphate (G-6-P) (67). Lactate dehydrogenase (LDH) is a crucial enzyme in glycolysis. LDH exists in three isoforms: Lactate dehydrogenase A (LDHA), Lactate dehydrogenase B (LDHB), and Lactate dehydrogenase C (LDHC). Among these, LDHA is the predominant isoform in various malignant tumors, including colon cancer, ovarian cancer, gastric cancer, and lung cancer (68). LDH catalyzes the conversion of pyruvate (PA) to lactic acid (LA), and elevated levels of lactic acid can lead to extracellular acidosis, which in turn promotes tumor cell invasion, angiogenesis, and metastasis (69).

Glabridin significantly inhibited the expression of glycolysis-related genes HK-2 and LDHA in melanoma cells, and reduced levels of lactate (LD) and ATP in the cell culture medium. Furthermore, animal studies demonstrated that glabridin intervention significantly slowed tumor tissue growth in mice, resulting in up-regulation of cellular Bax expression and down-regulation of B-cell lymphoma-2 (Bcl-2), HK-2, and LDHA expression in tumor tissues (37). During glycolysis, glucose transporter protein-1 (GLUT-1) is a key protein responsible for transporting glucose into the cell and maintaining the intracellular glucose concentration (70). Li et al. (71) found that glabridin significantly downregulated GLUT-1 expression and inhibited the glycolytic pathway in MDA-MB-231 breast cancer cells, regulating their energy metabolism. Therefore, the mechanism of apoptosis induced by glabridin may be related to its regulation of the expression of glycolysis-related genes.

Inhibition of the glycolytic pathway as a novel cancer therapeutic strategy has shown initial success in tumor models such as melanoma and breast cancer. Glabridin exhibits anti-tumor potential by modulating the glycolytic pathway and has demonstrated remarkable effects in several cell lines and animal models. However, the inhibition of glycolysis may be accompanied by metabolic adaptation and the development of drug resistance. Therefore, precisely regulating the glycolytic pathway to enhance therapeutic effects and prevent the development of drug resistance remains a key focus for future research.

#### 5.2.2 Promotion of mitochondrial apoptosis pathway

Mitochondria play a crucial role in regulating energy metabolism and apoptosis. In cancer cells, mitochondria overproduce reactive oxygen species (ROS), which contribute to cancer progression by inducing genomic instability, modifying gene expression, and participating in signaling pathways (72).

Yang et al. found that glabridin significantly inhibits the proliferation of bladder urothelial carcinoma cells and induces apoptosis (38). In a nude mouse model, glabridin significantly inhibits tumor growth and prolongs the survival time of tumor-bearing mice. The mechanism may involve glabridin-induced permeabilization of the outer mitochondrial membrane, which leads to the release of mitochondrial cytochrome c (CYCS) and subsequently triggers apoptosis. In addition, glabridin promotes vacuole formation in the cytoplasm of MDA-MB-231 breast cancer cells, causing loss of mitochondrial membrane potential and ROS production, leading to mitochondrial dysfunction and cancer cell apoptosis (73).

The role of glabridin in bladder and breast cancer cells reveals its potential to induce apoptosis through ROS production and loss of mitochondrial membrane potential. This mechanism may have broad applications in cancer therapy, as inducing cancer cell death through targeting the mitochondrial pathway is a promising strategy to overcome drug resistance. However, precisely regulating this process to avoid damage to normal cells remains a key focus for future research.

#### 5.2.3 Inhibition of the epidermal growth factor receptor signaling pathway

EGFR is a receptor tyrosine kinase commonly associated with cancer progression and poor prognosis. Inhibition of EGFR tyrosine kinase activity is considered a promising strategy for cancer treatment (74). Studies have shown that EGFR signaling is closely linked to breast cancer development and resistance to cytotoxic drugs (75).

Through molecular docking, ADMET profiling, and pharmacophore modeling, it was found that glabridin is an effective EGFR inhibitor, with an optimal binding affinity of -7.63 kcal/mol, comparable to the highly effective anti-cancer drug afatinib, and has demonstrated good efficacy in the treatment of breast cancer (76). Zhu et al. (77) found that glabridin can reduce the viability of breast cancer SK-BR-3 cells and induce apoptosis. Further studies showed that glabridin down-regulated the levels of phosphorylated epidermal growth factor receptor (P-EGFR) and phosphorylated protein kinase B (P-AKT) and promoted the expression of cysteine-aspartic acid protease 3 (caspase-3), cysteine-aspartic acid protease 8 (caspase-8), and cysteine-aspartic acid protease 9 (caspase-9), indicating that glabridin exerts its anti-cancer effects by regulating the activation of the EGFR signaling cascade. For detailed information, please refer to **Table 2**.

**Table 2:** Mechanism of glabridin-induced apoptosis in tumor cells.

Cancers	Effective concentrations	Cell type/Animal model	Possible mechanisms	Reference
Melanoma	20 mg/kg	C57BL/6 mice	Glabridin induces tumor cell apoptosis by upregulating the pro-apoptotic gene Bax and downregulating the anti-apoptotic gene Bcl-2 while inhibiting glycolysis by reducing HK2 and LDHA expression.	(37)
	20, 40, 60, 80, 100 μM	Mouse melanoma cells: B16F10		
Breast cancer	5, 10, 20, 30 μM	Human breast cancer cell lines: MDA-MB-231	Glabridin reduces glucose uptake and energy supply in breast cancer cells by downregulating GLUT1 expression. It inhibits LDH activity and lactic acid production, blocks glycolysis, and lowers ATP levels, restricting cancer cell growth and proliferation.	(71)
Urothelial bladder carcinoma	10 mg/kg	Nude-mice	Glabridin upregulates pro-apoptotic proteins like CYCS, activating the intrinsic apoptosis pathway and inducing cell apoptosis.	(38)
	13.4, 18.6 μM	Urothelial bladder carcinoma cell: BIU-87, EJ		
Breast cancer	20, 40, 60, 80, 100 μM	Human breast cancer cell lines: MDA-MB-231, MCF7	Glabridin upregulates endoplasmic reticulum stress markers (CHOP), inducing stress and protein aggregation that disrupts cancer cell metabolism. It also increases ROS production and lowers mitochondrial membrane potential, leading to oxidative stress, impaired mitochondrial function, and cell death.	(73)
Breast cancer	10, 50, 100 μM	Breast carcinoma cell lines: SK-BR-3	Glabridin activates the apoptosis pathway by upregulating caspases 3, 8, and 9 to reduce cell viability; it inhibits phosphorylated EGFR, and p-AKT, blocking breast cancer proliferation signals; and lowers intracellular ROS levels, reducing oxidative stress and inhibiting tumor progression.	(77)

### 5.3 Inhibition of tumor cell proliferation

**Figure 5:**
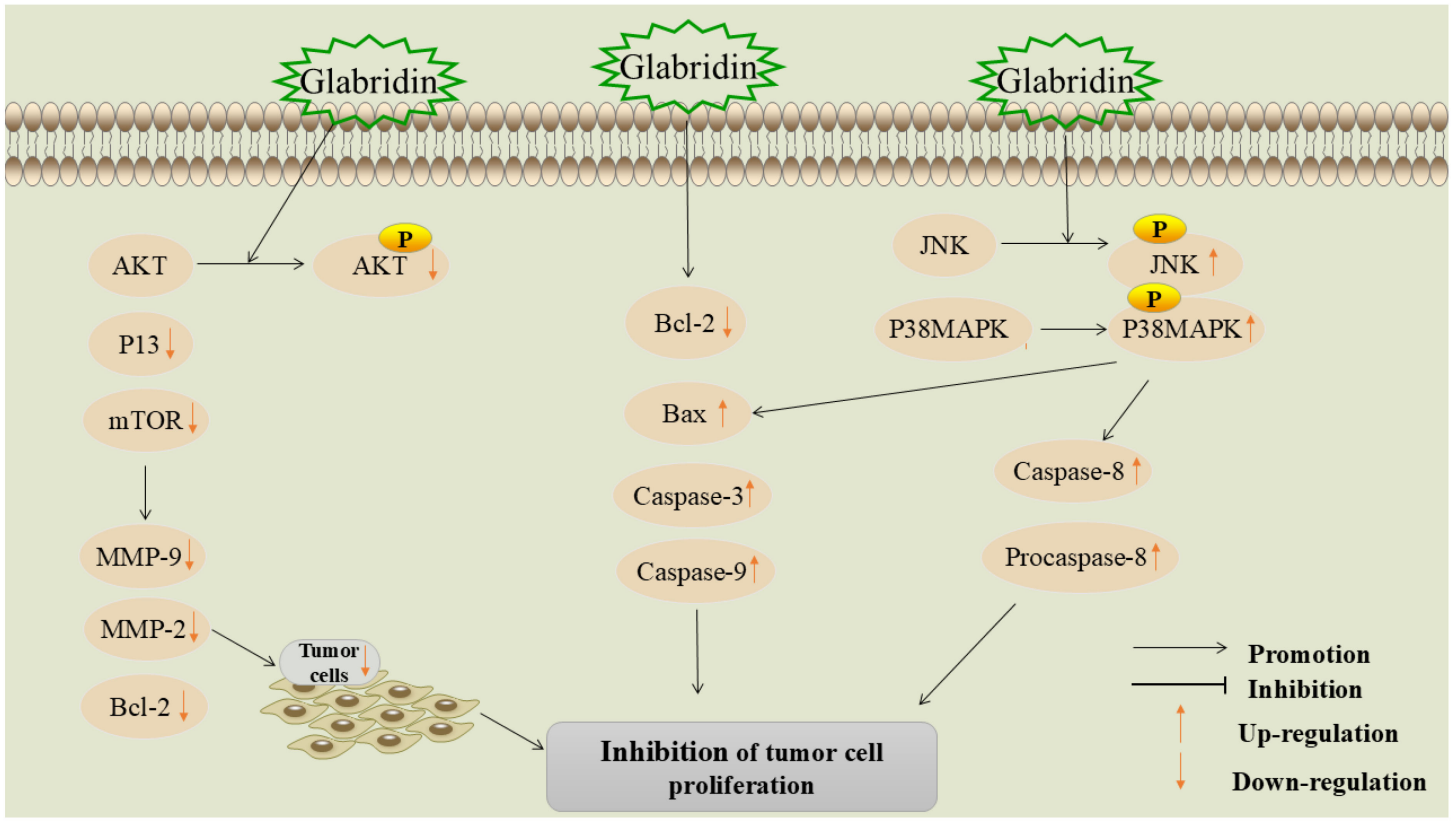
Mechanism of glabridin suppressing proliferation in tumor cells.

Cell proliferation is a crucial factor in cell growth and differentiation. Inhibiting tumor cell proliferation can effectively hinder cancer progression and is a critical aspect of cancer treatment (64, 78). The mechanism of glabridin inhibiting tumor cell proliferation is shown in **Figure 5**.

#### 5.3.1 Inhibition of P13K/Akt signaling pathway

The phosphoinositide 3-kinase/protein kinase B (PI3K/Akt) signaling pathway is a critical signaling pathway in various cancers, exerting extensive regulatory effects on cell survival, growth, migration, metabolism, and angiogenesis (79).

Tan et al. (80) found that glabridin significantly inhibits the proliferation of prostate cancer cells. Network pharmacology and molecular docking results showed that the PI3K/Akt pathway is key to this process. *In vitro* experiments showed that glabridin significantly inhibits Akt phosphorylation, suggesting it inhibits prostate cancer cell proliferation by regulating the PI3K/Akt pathway. Zhang et al. (81) also confirmed this view in their study, finding that glabridin promoted ROS signaling in prostate cancer cells in a dose-dependent manner. The excessive production of ROS is an important marker of cancer and can regulate a variety of tumor-related signaling pathways (82). Glabridin can promote apoptosis by inhibiting PI3K/Akt signaling via ROS, enhancing caspase-3 activity, and increasing the Bcl-2-associated X protein (Bax)/Bcl-2 expression ratio.

The mammalian target of rapamycin (mTOR) is a protein kinase that regulates cell proliferation, survival, metabolism, and immunity. It plays an important role in many human cancers and can promote tumor cell growth and metastasis through multiple mechanisms (83). Li et al. (84) investigated the effect and molecular mechanism of glabridin on inhibiting the proliferation of colon cancer cells. After glabridin intervention, the expression of PI3K, Akt, and mTOR proteins, as well as downstream molecules MMP-9, MMP-2, and Bcl-2, was significantly reduced, while the expression of Bax, caspase-3, and caspase-9 proteins was significantly increased. The mechanism of action may be related to the inhibition of the PI3K-Akt-mTOR signaling pathway.

#### 5.3.2 Inhibition of JNK/P38 signaling pathway

JNK is a member of the mitogen-activated protein kinase (MAPK) family (85) and plays a role in regulating signaling pathways involved in tumor cell proliferation, migration, and apoptosis. It is regarded as a potential target for cancer therapy (86). p38 MAPK is also a member of the MAPK family and can be activated by inflammatory factors and various environmental stresses. These kinases are key components of signal transduction cascades used by cancer cells to sense and adapt to their environment (87).

Studies have found that glabridin regulates JNK1/2 and P38 MAPK phosphorylation, downregulates the anti-apoptotic protein Bcl-2 and procaspase-9, and upregulates Bax and caspase-3, inhibiting colorectal cancer cell proliferation (88). Huang et al. investigated the molecular mechanism of glabridin’s anti-cancer effects in human promyelocytic leukemia and found (89) that glabridin upregulates the phosphorylation of P38 MAPK and JNK1/2 in a time- and dose-dependent manner, promoting the activation of caspase-3, caspase-8, and caspase-9, thereby inhibiting the proliferation of acute myeloid leukemia (AML) cell lines (HL-60, MV4-11, U937, and THP-1). For detailed information, please refer to **Table 3**.

**Table 3:** Mechanism of glabridin suppressing proliferation in tumor cells.

Cancers	Effective concentrations	Cell type	Possible mechanisms	Reference
Prostate cancer	72 μM	Prostate cancer cell line: PC-3	Glabridin induces apoptosis by inhibiting AKT phosphorylation.	(80)
Prostate cancer	5, 10, 20 μM	Prostate cancer cell lines: DU-145, LNCaP	Glabridin increases intracellular and mitochondrial ROS levels, reduces the p-Akt/Akt ratio, inhibits cell proliferation, and induces apoptosis by upregulating Bax and caspase-3 and downregulating Bcl-2.	(81)
Colon cancer	12.5, 25, 50, 100 μM	Colon cancer cell lines: SW480, SW620, HT29 and HCT116	Glabridin inhibits cancer cell proliferation by downregulating PI3K, AKT, and mTOR, upregulating Bax, caspase-3, and caspase-9, and reducing Bcl-2, inducing colon cancer cell death through the intrinsic apoptosis pathway.	(84)
Colon cancer	1, 5, 10, 25 μM	Human colorectal cancer cell lines: RKO	Glabridin enhances tumor cell apoptosis by upregulating pro-apoptotic proteins Bax and Caspase-3 and downregulating anti-apoptotic proteins Bcl-2 and Procaspase-9.	(88)
Acute myeloid leukemia	10, 20, 40 μM	Acute myeloid leukemia cell lines: HL-60, MV4-11, U937 and THP-1	Glabridin increases intracellular and mitochondrial ROS production, downregulates Akt and ERK1/2 phosphorylation to inhibit tumor cell proliferation, and upregulates p38 and JNK phosphorylation, increasing Bax, activating caspase-3 and PARP cleavage, and decreasing Bcl-2 to enhance apoptosis.	(89)

### 5.4 Induction of tumor cell cycle arrest

The disruption of normal cell cycle progression is a fundamental mechanism underlying tumorigenesis. Interfering with and blocking various stages of the cell cycle can effectively inhibit the division and proliferation of tumor cells (90). Cyclins regulate the cell cycle by binding to and activating cyclin-dependent kinases (CDKs) (91). Wang et al. found that glabridin can significantly inhibit the expression of cyclin-dependent kinase 2 (CDK2), cyclin-dependent kinase 4 (CDK4), and G1/S-specific cyclin D3(cyclin D3), blocking the cell cycle at the G1 phase, thereby inhibiting the proliferation of liver cancer cells (92).

The Wnt/β-catenin signaling pathway and its downstream target proteins are crucial regulators of cell proliferation (93). Abnormal Wnt/β-catenin signaling leads to the proliferation and differentiation of cancer cells, playing a significant role in tumorigenesis and progression (94, 95). Huang et al. found that glabridin exhibits high cytotoxicity against cervical cancer cells (Hela), promoting cell cycle arrest in the S and G2/M phases and inducing apoptosis in the G0/G1 phase (96). Experimental results showed a significant reduction in the expression levels of Wnt1, β-catenin, G1/S-specific cyclin D3(cyclin D1), and MMP-2 proteins. This mechanism may be associated with the inhibition of the Wnt/β-catenin signaling pathway.

### 5.5 Induction of autophagy in tumor cells

Autophagy is an intracellular catabolic process that involves the delivery of misfolded proteins, damaged or aged organelles, and excess cytoplasmic components to lysosomes for degradation via autophagosomes. This process is crucial for maintaining cellular homeostasis and vitality (97). Microtubule-associated protein light chain 3-II (LC3-II) and Beclin 1, a key molecule in autophagy, are autophagy-related markers that play crucial roles in the autophagic process (98, 99).

Hsieh et al. investigated the role of glabridin in inducing autophagy and related signaling pathways in liver cancer cells. They found that glabridin upregulated the expression of LC3-II and Beclin1 and induced autophagy by modulating the phosphorylation of p38MAPK and JNK1/2, which subsequently led to cell death (100).

### 5.6 Inhibition of tumor angiogenesis

Angiogenesis is a critical marker of cancer development and progression (101). The rapid proliferation of malignant tumors leads to insufficient oxygen and nutrient supply, driving tumor cells to promote angiogenesis by increasing angiogenic factors like vascular endothelial growth factor (VEGF), platelet-derived growth factor (PDGF), and angiopoietin. This process supports the survival, growth, and proliferation of tumor cells and facilitates tumor invasion and metastasis to other tissues via the bloodstream (102). Consequently, inhibiting tumor angiogenesis has emerged as a significant strategy in cancer treatment.

**Figure 6:**
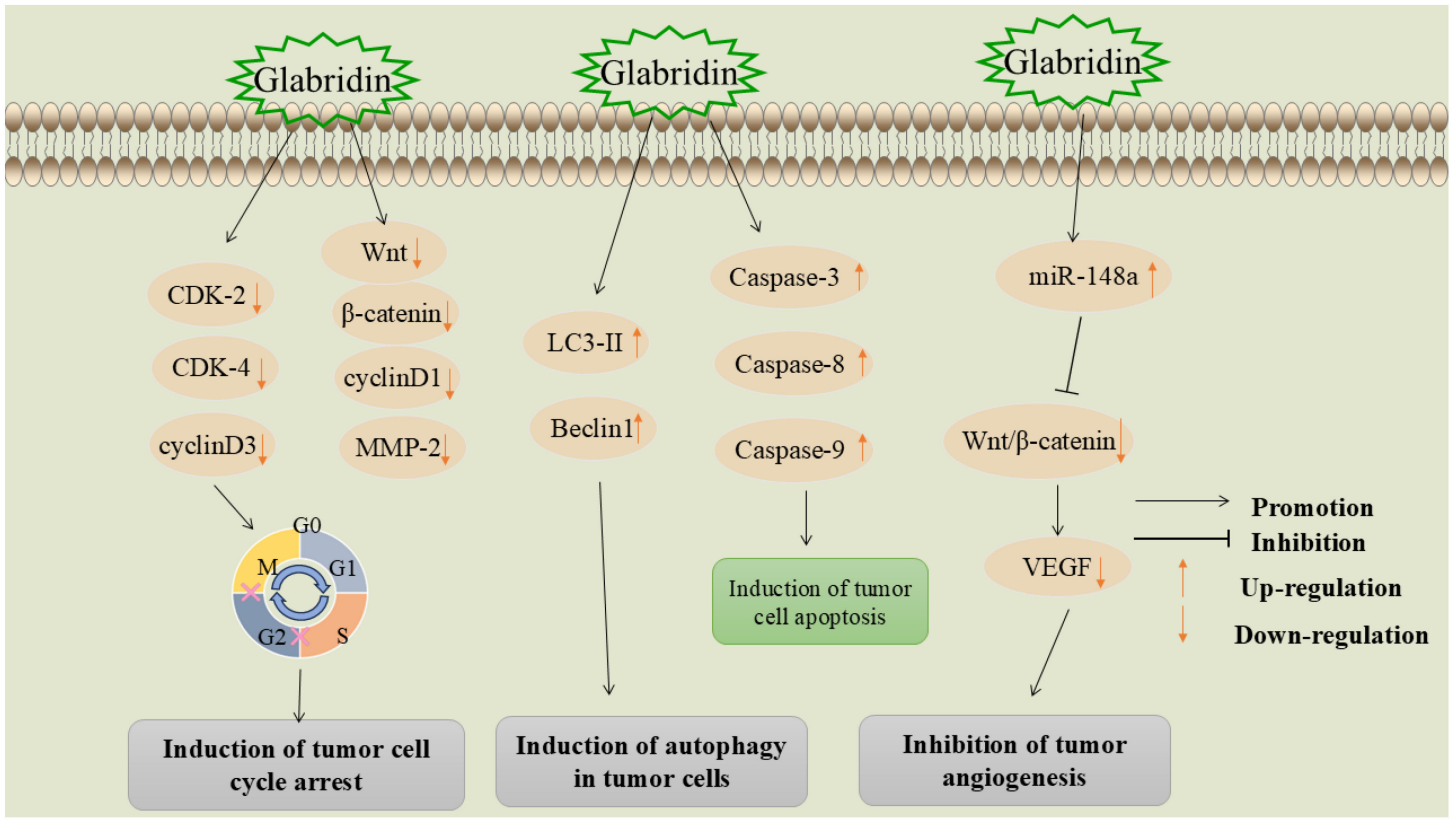
Mechanisms by which glabridin induces cellular autophagy, induces cell cycle arrest, and inhibits tumor angiogenesis.

Mu et al. (103) found that the molecular mechanism of glabridin in treating breast cancer involves upregulating miR-148a and inhibiting the Wnt/β-catenin pathway. Specifically, glabridin upregulates miR-148a expression in a dose-dependent manner. miR-148a targets Wnt1, leading to the accumulation of β-catenin in the cell membrane and nucleus. This results in reduced VEGF secretion, decreased angiogenesis, and ultimately inhibits tumor growth and development. The mechanism by which glabridin induces cellular autophagy, causes cell cycle arrest, and inhibits tumor angiogenesis is shown in **Figure 6**.

### 5.7 Enhancing chemotherapy drug sensitivity

The conventional method for treating malignant tumors is chemotherapy; however, cancer cells are prone to developing resistance to these drugs, which often leads to reduced efficacy or even treatment failure (104). Combination chemotherapy is a treatment method that involves the simultaneous use of two or more chemotherapy drugs. Compared with monotherapy, combination therapy not only reduces drug resistance but also more effectively controls and cures cancer (105).

5-fluorouracil is one of the most commonly used chemotherapeutic agents and is widely employed in the treatment of colon cancer, breast cancer, and liver cancer (106). However, its low bioavailability, short half-life, rapid metabolism, and the development of resistance following chemotherapy limit its therapeutic efficacy (107). Zhang’s research indicates that glabridin, in combination with 5-fluorouracil, can effectively inhibit the malignant proliferation and invasion of the MKN-45 gastric cancer cell line (108). This effect may be associated with the downregulation of MMP-9 and MMP-2 expression.

Tamoxifen is an anti-estrogen drug that has become the primary treatment option for postmenopausal women with metastatic breast cancer (109). Paclitaxel is a naturally occurring compound found in yew trees and is widely used for cancer treatment (110). The current drug resistance of cancer cells has become a major obstacle to the effective treatment of these two drugs (111, 112). Lin et al. (113) found that glabridin significantly enhanced the anti-proliferative and pro-apoptotic effects of tamoxifen on breast cancer cells, as well as the anti-proliferative and pro-apoptotic effects of paclitaxel on prostate cancer cells. The mechanism may be related to a reduction in mitochondrial transmembrane potential and an increase in intracellular ROS, which in turn induces the activation of the caspase cascade.

Doxorubicin (DOX) is a topoisomerase inhibitor and an anthracycline-class anticancer drug (114). Qian et al. (115) found that glabridin reduces the IC50 values of paclitaxel and DOX in MDA-MB-231 breast cancer cells and enhances DOX-induced apoptosis. The mechanism involves glabridin downregulating the expression of P-glycoprotein (P-gp) and competitively inhibiting P-gp efflux pumps, thereby increasing the accumulation of DOX and paclitaxel in breast cancer cells. In another study, researchers assessed the cytotoxicity of glabridin on three cancer cell lines: A2780 (human ovarian carcinoma), SKNMC (human neuroblastoma), and H1299 (human non-small cell lung carcinoma). The results indicated that glabridin can kill up to 90% of cancer cells in these lines, with IC50 values of 10, 12, and 38 μM for A2780, SKNMC, and H1299, respectively. Additionally, glabridin enhances the cytotoxicity of DOX against these cancer cells and significantly reduces cell viability. The synergistic inhibitory effect of glabridin and DOX on H1299 cells was most pronounced, with glabridin significantly increasing DOX accumulation in a dose-dependent manner. At DOX concentrations of 15 µM and 30 µM, intracellular accumulation of doxorubicin increased by 2.8-fold and 2.5-fold, respectively (116).

Glabridin not only enhances the anti-tumor effects of DOX but also reduces DOX-induced cardiotoxicity. Huang et al. (117) found that glabridin can reduce DOX-induced myocardial enzyme leakage, including aminotransferase, creatine kinase, lactate dehydrogenase, and creatine kinase-MB, downregulate pro-apoptotic proteins (Bax, caspase 9, and caspase 3), and upregulate anti-apoptotic proteins (Bcl-2) in cardiac tissues. In addition, glabridin can regulate the DOX-induced imbalance of intestinal flora, thereby reducing the ratio of M1/M2 macrophages in the colon. This is accompanied by the downregulation of lipopolysaccharide (LPS) in feces and peripheral blood, and the upregulation of butyrate, indicating that glabridin can effectively prevent DOX-induced cardiotoxicity by modulating the intestinal flora and polarization of colonic macrophages. For detailed information, please refer to **Table 4**.

**Table 4:** Mechanisms by which glabridin induces cellular autophagy, induces cell cycle arrest, inhibits tumor angiogenesis and enhances chemotherapy drug sensitivity.

Pharmacological action	Cancers	Effective concentrations	Cell type	Possible mechanisms	Reference
Induction of tumor cell cycle arrest	Liver cancer	0, 15, 35, 45 μM	Hepatoma carcinoma cell lines: HepG2	Glabridin inhibits cancer cell proliferation and survival by downregulating cyclin D3, CDK2, and CDK4, blocking the G1/S phase transition, and reducing CREB and ATF1 phosphorylation.	(92)
	Cervical cancer	1, 2.5, 5, 10, 20 μM	Cervical cancer cell lines: Hela	Glabridin downregulates Wnt1, β-catenin, and downstream proteins Cyclin D1, MMP-2, and Survivin, inhibiting the tumor cell cycle.	(96)
Induction of autophagy in tumor cells	Liver cancer	25, 50, 100 μM	Human hepatoma cell lines: Huh7, HepG2 and Sk-Hep-1	Glabridin induces tumor cell apoptosis by upregulating caspase-3, caspase-8, caspase-9 cleavage while enhancing autophagy by upregulating LC3-II and beclin-1.	(100)
Inhibition of tumor angiogenesis	Breast cancer	10 μM	Human breast cancer cell lines: MDA-MB-231 and Hs-578T	Glabridin upregulates miR-148a, inhibits Wnt1 and β-catenin activity, and reduces the expression of proliferation and angiogenesis genes such as VEGF.	(103)
Enhancing chemotherapy drug sensitivity	Gastric cancer	6, 12, 25, 30, 40 µM	Human breast cancer cell lines: MDA-MB-231	Glabridin promotes tumor cell apoptosis by upregulating BAX, Caspase-3, Caspase-8, and Caspase-9 and downregulating Bcl-2, and inhibits cancer cell invasion and metastasis by downregulating N-Cadherin and upregulating E-Cadherin to reduce cell adhesion.	(108)
	Endometrial cancer	50 μM	Human breast cancer cell lines: MDA-MB-231	Glabridin reduces tumor invasiveness by inhibiting cell migration and EMT through upregulating E-cadherin and downregulating β-catenin.	(113)
	Breast cancer	10, 30 μM	Breast cancer cell lines: MDA-MB-231	Glabridin increases DOX accumulation in MDR cells by inhibiting P-gp expression and ATPase activity, enhancing its cytotoxicity and apoptosis effects.	(115)
	Ovarian cancer, Lung cancer	5, 10, 15, 20, 25, 30 µM	Human euroblastoma cells: SKNMC, Human ovarian carcinoma cell lines: A2780, and human non-small cell lung carcinoma cells: H1299	Glabridin induces apoptosis by upregulating Bax, downregulating Bcl-2, and activating caspase-3.	(116)

### 5.8 Glabridin prevents skin and endometrial cancer

#### 5.8.1 Prevention of skin cancer

Ultraviolet B (UVB) is considered a major environmental factor detrimental to human health. It can activate various signal transduction pathways and induce the expression of multiple specific genes, which are the main mechanisms behind skin aging and cancer development (118). Therefore, it is important to prevent and reverse UVB damage to reduce the incidence of skin cancer. A study on the anti-photoaging properties of glabridin found that glabridin effectively attenuated the levels of pro-inflammatory factors, including IL-1β, tumor necrosis factor-α (TNF-α), IL-22, and IFN-γ, thereby preventing UVB-induced photoaging in mice and reducing skin inflammation (119). Another study found that glabridin possesses antioxidant activity, can prevent DNA oxidative damage caused by UVB irradiation, and reduces the production of ROS and the activation of apoptosis pathway proteins (120), thereby preventing skin cancer.

#### 5.8.2 Prevention of endometrial cancer

It has been reported that the affinity of glabridin for the human estrogen receptor is comparable to that of genistein, the best-known phytoestrogen (22). Abnormally high estrogen levels are associated with an increased incidence of certain cancers, especially breast and endometrial cancer. The International Agency for Research on Cancer (IARC) has classified estrogen and combined estrogen-progestin postmenopausal therapy as known human carcinogens (23). Glabridin exhibits an estrogenic effect in the endometrial cell line Ishikawa, increasing in a dose-dependent manner and potentially reducing the risk of endometrial cancer (121). Another study found that glabridin, in combination with tamoxifen, exhibited estrogenic activity and inhibited cell proliferation (122). Compared to tamoxifen alone, glabridin in combination with tamoxifen reduced the proliferation of MCF-7 cells twofold. This suggests that combining glabridin with tamoxifen may serve as an estrogen replacement therapy to reduce the risk of tamoxifen-associated endometrial cancer.

## 6 Glabridin drug delivery systems

**Figure 7:**
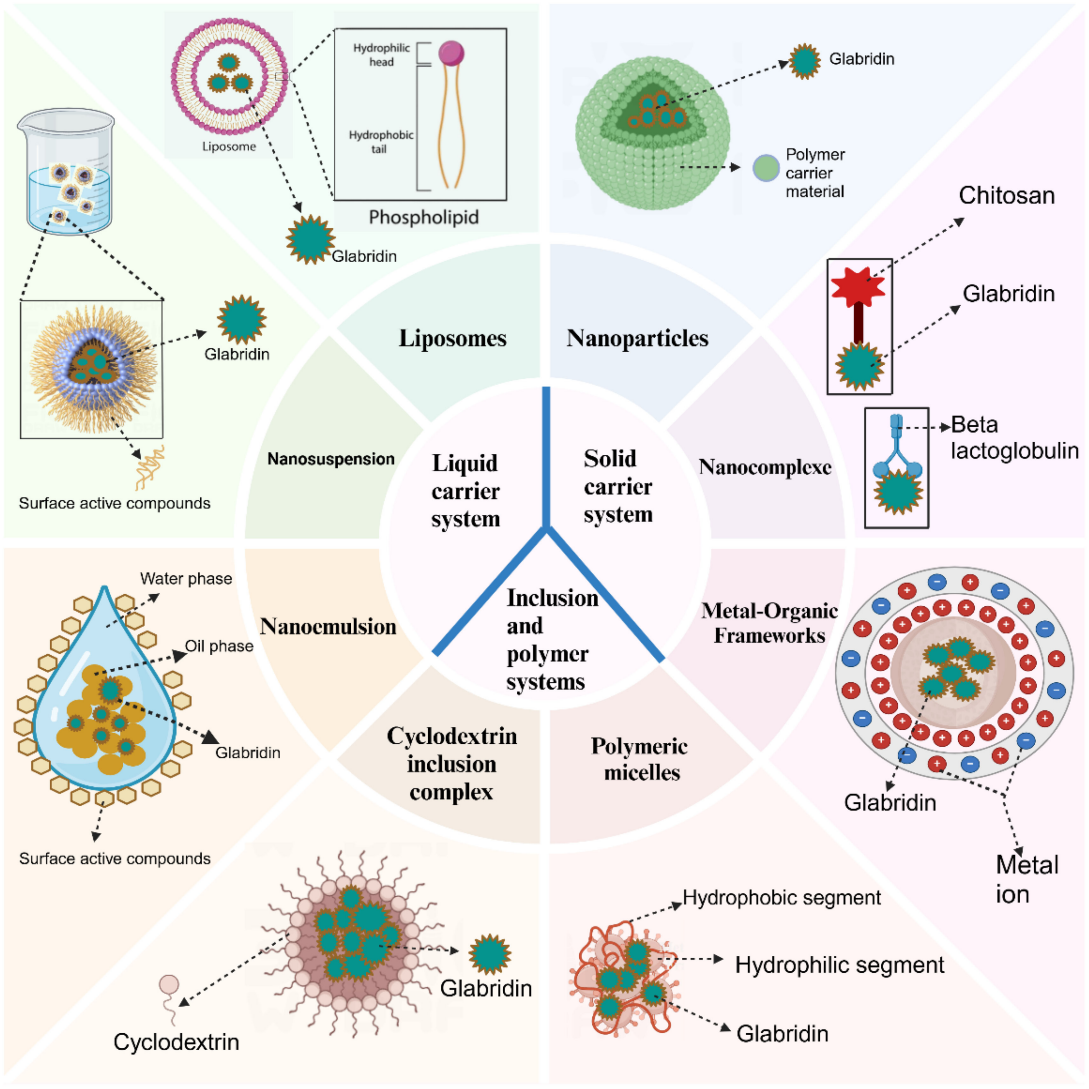
Gabridin’s drug delivery system.

Glabridin exhibits inhibitory effects on the growth of various tumors; however, its poor water solubility, low bioavailability, and lack of specific targeting limit its clinical application. Numerous studies have demonstrated that novel drug delivery systems can significantly enhance the solubility, stability, targeting, and bioavailability of poorly soluble drugs (123). Currently, several glabridin drug delivery systems have been developed, such as liposomes, cyclodextrin inclusion complexes, nanoparticles, and polymer micelles. These advancements offer promising solutions for improving the clinical application of glabridin. Glabridin drug delivery systems can be classified based on the physical state of the delivery system and the mechanisms of drug release as follows: The glabridin drug delivery system is shown in **Figure 7**.

### 6.1 Liquid carrier system

#### 6.1.1 Liposomes

Liposomes are vesicular structures formed by encapsulating drugs within a lipid bilayer (124). They can significantly enhance the permeability and solubility of drugs and possess high biocompatibility, non-immunogenicity, low toxicity, and biodegradability (125, 126). Thus, they serve as an excellent drug delivery system.

Huang et al. prepared glabridin liposomes using the complex coacervation method and investigated their particle size distribution, *in vitro* drug release characteristics, and stability. The results showed that glabridin liposomes had superior *in vitro* drug release compared to pure glabridin in the first 120 minutes. After three months, the mass fraction of glabridin in the liposomes remained at 38.5%, with a retention rate of 96.25% (127). Zhang et al. prepared glabridin liposomes using the thin film dispersion method and evaluated their effects on skin aging. The results demonstrated that glabridin liposomes exhibited lower cytotoxicity compared to pure glabridin and were more effective in inhibiting melanin production (128). Another study employed the ultrasonic emulsification method to prepare glabridin nanoliposomes. The results indicated that glabridin nanoliposomes exhibited excellent sustained release, transdermal absorption, and photostability, thereby enhancing the utilization of glabridin (129).

Wang et al. utilized Confocal Raman Spectroscopy (CRS) to assess the epidermal permeability of glabridin liposomes compared to pure glabridin. The study showed that glabridin liposomes had 3.8 times greater skin permeability than pure glabridin, and liposome encapsulation significantly enhanced its transdermal absorption and bioavailability (130).

Currently, liposomes are regarded as one of the most advanced drug delivery vehicles. Given their excellent biocompatibility and low toxicity, they have been successfully utilized in various small-molecule drugs and large-molecule biologics (131). For glabridin, liposomes significantly enhance its skin penetration and antioxidant activity. However, the significant efficacy of glabridin liposomes in skin cancer requires further experimental verification. Additionally, the production processes of liposomes (e.g., thin-film dispersion and ultrasonic emulsification) have matured (132) and received multiple approvals from the US Food and Drug Administration (FDA) and the European Medicines Agency (EMA) (133), particularly for cancer treatment drugs (134). Therefore, glabridin liposomes are regarded as one of the most promising delivery systems for rapid advancement to clinical applications. However, their high material cost and the necessity of refrigeration post-preparation, owing to the susceptibility of phospholipids to oxidation, limit their long-term stability (135).

#### 6.1.2 Nanosuspension

Nanosuspension is the dispersions of drug nanoparticles within a liquid medium, typically water, and consists mainly of nanoparticles with sizes ranging from 100 to 1000 nm. To maintain stability, nanosuspensions are generally stabilized with a small amount of surfactant (136, 137).

Wang et al. prepared a glabridin nanosuspension using the anti-solvent precipitation-homogenization method and optimized particle size by evaluating formulation parameters with the Box-Behnken design (138). The optimal formulation consisted of 0.25% glabridin, 0.47% poloxamer 188, and 0.11% polyvinylpyrrolidone K30. The average particle size of the resulting nanosuspension was 149.2 nm with a polydispersity index of 0.254. The results showed that the nanosuspension significantly enhanced the transdermal penetration flux of glabridin both *in vitro* and *in vivo*, without any lag phase. After 3 months of storage at room temperature, no significant particle aggregation was observed, and the drug loss was minimal at 5.46%.

Due to the large specific surface area of nanoparticles, nanosuspensions can enhance the stability of drugs in solvents and extend their shelf life (139, 140); they are suitable for topical administration (e.g., eyes, lungs, and skin) (141). Nanosuspensions are relatively inexpensive to produce and simple to scale up. Several nanosuspension formulations have been approved worldwide (142). However, particle aggregation and changes in particle size need to be controlled during long-term storage.

#### 6.1.3 Nanoemulsion

Nanoemulsion is a thermodynamically unstable yet kinetically stable colloidal solution, formed by dispersing droplets with a particle size of less than 100 nm in a different liquid medium. Its composition typically includes water, oil, surfactants, and co-surfactants (143). Nanoemulsions are characterized by low surface tension, good physical stability, high solubility, and ease of preparation (144).

Liu et al. prepared nanoemulsions using various oil phases and compared their physicochemical properties, including apparent solubility, droplet size, and zeta potential, as well as *in vitro* and *in vivo* skin permeability and stability (145). Their study identified the eutectic mixture of menthol and camphor as the optimal solvent for glabridin. The solubility of glabridin in the prepared nanoemulsion was 76.6 mg/g, and both its *in vitro* and *in vivo* skin permeability were significantly enhanced compared to pure glabridin. Additionally, the nanoemulsion exhibited excellent chemical stability.

Nanoemulsions are highly efficient transdermal absorption systems suitable for local drug delivery (146). For glabridin, Nanoemulsion can enhance its skin absorption rate and stability; however, whether they can improve glabridin’s efficacy against skin cancer requires further experimental verification. The production processes of Nanoemulsion are relatively simple and easy to scale up (147). Currently, many approved products in cosmetics and pharmaceuticals utilize Nanoemulsion (141). However, the chemical stability of Nanoemulsion requires improvement, as they may undergo phase separation at extreme temperatures (high or low) (148).

### 6.2 Solid carrier system

#### 6.2.1 Nanoparticles

Nanoparticles are solid particles sized between 10 and 1000 nm, created using nanotechnology to dissolve or encapsulate drugs within a polymer carrier material (149). These nanoparticles can target drug delivery to tumors or diseased tissues (150), exhibiting high drug loading, high encapsulation efficiency, and controlled drug release (151).

Li et al. discovered that glabridin-loaded nanoparticles not only possess antioxidant activity but also specifically accumulate in the spleen to inhibit inflammatory responses (12). Chen et al. employed an ionic-gelation method to prepare glabridin nanoparticles by blending glabridin with chitosan (CS) and poly-γ-glutamic acid (γ-PGA). The results indicated that the appearance and particle size of the nanoparticles remained stable after 90 days, with an encapsulation rate of 88.30 ± 0.54% and a drug loading rate of 26.47 ± 0.73% (152).

The preparation processes of nanoparticles are well-established, with methods such as solvent evaporation and ultrasound widely applied in industrial production (153). Currently, various nanoparticle drugs have been approved for cancer treatment (154), and their high targeting and controlled release properties demonstrate significant potential in clinical trials for cancer therapies. However, different types of nanomaterials exhibit significant variability in toxicity and metabolic pathways, which may lead to potential immune responses and cumulative toxicity; therefore, rigorous safety evaluations are still required for clinical applications (155).

#### 6.2.2 Nanocomplex

Nanocomplexes are nano-aggregates formed by non-covalent interactions between carrier molecules, such as proteins or oligosaccharides, and drugs (156), which can improve the physicochemical properties of drugs, increasing solubility, encapsulation rate, and bioavailability (157).

Chitosan is a natural polysaccharide widely utilized in drug delivery applications owing to its excellent biocompatibility and biodegradability (158). Su et al. studied the stability of glabridin in the chitosan-glabridin nanocomplex, finding that the encapsulation rate of glabridin in the nanocomplex could reach up to 84%, significantly enhancing stability in aqueous solution and under ultraviolet light (159). Beta-lactoglobulin (β-Ig) is an essential milk protein widely utilized in food and drug delivery systems owing to its excellent biocompatibility and biodegradability (160). Based on the fact that β-lg readily solubilizes in water and binds many small hydrophobic molecules, Wei et al. developed a novel nanocomplexed glabridin with β-lg using an antisolvent precipitation method (161). Molecular docking modeling showed that glabridin binds to β-lg through hydrophobic and hydrogen bond interactions, increasing its solubility in aqueous solution by 21 times. At the same concentration, nanocomplexed glabridin with β-lg shows better 2,2-diphenyl-1-pyridinium hydrazone and 2,2’-azino-3-ethylbenzothiazoline-6-sulfonic acid radical scavenging capacities compared to pure glabridin. Therefore, the nanocomplexation of β-lg with glabridin, which enhances the solubility of glabridin in aqueous systems, offers a promising opportunity for β-lg to serve as an effective carrier molecule.

Nanocomplexes enhance drug stability and solubility, effectively preventing rapid metabolism or degradation within the biological environment (162). They perform excellently in localized and targeted drug delivery, demonstrating significant potential for cancer treatment (163). However, the long-term stability of nanocomplexes *in vivo* and their potential immune response risks require further investigation.

#### 6.2.3 Metal-organic frameworks

Metal-organic frameworks (MOFs) are porous materials formed by the coordination of metal ions or metal clusters with organic ligands. Compared to other nanomaterials, MOF nanoparticles have several advantages, including adjustable pore size, low density, and high surface area (164, 165). These characteristics have led to the increasing use of MOFs as carriers in drug delivery systems in biomedical research, particularly for the delivery of anticancer drugs (166).

ZIF-8 is a special metal-organic framework formed by the self-assembly of zinc ions (Zn^2+^ >) and 2-methylimidazole (2-MIM). In addition to the advantages of other MOFs, ZIF-8 shows high stability in aqueous solutions due to the strong interaction between Zn^2+^ and 2-MIM. Chen et al. prepared a ZIF-8 metal-organic framework (MOF) encapsulating glabridin using the anti-solvent precipitation method (167). The drug encapsulation efficiency was 98.67%, and pH-controlled release of glabridin was achieved. Additionally, cell experiments demonstrated that the MOF significantly enhanced the antioxidant activity, melanin-inhibiting activity, and bioavailability of glabridin.

MOFs can respond to external stimuli (e.g., temperature, light, pH) by adjusting their pore structures to enable controlled drug release (168), demonstrating significant potential in targeted drug delivery, gene therapy, and related fields. However, the incorporation of metal components raises concerns about the potential biotoxicity and biodegradability of MOFs, which remain insufficiently verified (169), posing challenges for safety assessments in biomedical applications.

### 6.3 Inclusion and polymer systems

#### 6.3.1 Cyclodextrin inclusion complex

An inclusion complex is a special complex formed when a drug is partially or completely encapsulated within the cavity structure of a host molecule (170). Cyclodextrin is a cyclic oligosaccharide synthesized by cyclodextrin glucosyltransferase (CGRase) using starch as a substrate (171). Its hydrophilic outer surface and relatively hydrophobic central cavity can form complexes with drugs, thereby enhancing their solubility and stability (172, 187).

Wei et al. employed a co-evaporation method to prepare a complex of glabridin and hydroxypropyl-β-cyclodextrin (HP-β-CD). The results demonstrated that at 25°C, the solubility of the complex in pure water was 40.74 ± 0.44 mM, which was 1852 times higher than that of glabridin. *In vitro* experiments revealed that the DPPH free radical scavenging capacity and the tyrosinase inhibitory activity of the complex were enhanced by approximately 9 and 20 times, respectively (173).

Li et al. demonstrated that the encapsulation efficiency and drug loading capacity of the glabridin/HP-β-CD inclusion complex were 90.03% and 14.51%, respectively. The saturation solubility of the inclusion complex was 109.36 mg/mL. The cumulative dissolution rates in gastric and intestinal juices after 1 hour were 15.75 and 12.4 times higher than those of glabridin, respectively, while the cumulative release rate over 24 hours was 53 times that of glabridin. The uptake of Caco-2 cells was 0.349 mg/g, significantly higher than that of glabridin (0.039 mg/g). These results indicate that the glabridin/HP-β-CD inclusion complex significantly enhances the dissolution and release of glabridin, thereby increasing its bioavailability (174). Thus, the glabridin/HP-β-CD inclusion complex is expected to be an ideal form of glabridin for drug delivery.

Wang et al. developed a novel inclusion complex, sulfobutylether-β-cyclodextrin/glabridin, using the freeze-drying method. The solubility of this inclusion complex was 1889 times higher than that of glabridin. Moreover, this inclusion complex exhibited excellent biocompatibility, along with significant antibacterial, anti-inflammatory, and antioxidant activities, and effectively accelerated the healing of skin damage (175).

Yao et al. prepared 2-sulfobutyl-β-cyclodextrin/glabridin inclusion complexes using freeze-drying, spray-drying, and kneading methods. The saturation solubility of these complexes was greater than 83 mg/mL. The encapsulation efficiency and drug loading capacity of the inclusion complex prepared by freeze-drying were 86.09% and 22.39%, respectively. The cumulative dissolution rates in gastric and intestinal fluids, as well as the antiproliferative activity against the human liver cancer cell line (HepG-2), were significantly higher than those of glabridin (176).

Cyclodextrin inclusion complex significantly enhances the solubility of glabridin and effectively improves the bioavailability of drugs, making them suitable for oral and topical administration (177). However, the production costs of cyclodextrin complexes remain relatively high (178). Studies indicate that cyclodextrin complexes are valuable not only for cancer and inflammation treatment but also for delivering novel small molecule and peptide drugs (133). Nonetheless, further toxicological and biocompatibility studies are required to ensure their safety across various administration routes (179, 180).

#### 6.3.2 Polymeric micelles

Polymeric micelles are thermodynamically stable colloidal solutions that form through the self-assembly of synthetic amphiphilic block copolymers in aqueous environments. Their hydrophilic shell and nanoscale particle size facilitate drug release within the body and enhance drug concentration at the target site (181).

Partially myristoylated chitosan pyrrolidone carboxylate (PMCP) is a cationic, amphiphilic derivative of chitosan. Senio et al. employed PMCP emulsification to develop novel, nanoparticle-sized cationic polymeric micelles encapsulating glabridin (182). The skin permeability and melanin inhibition efficacy of the polymeric micelles were evaluated using a human skin model. The results showed that the glabridin dose absorbed 24 hours after applying polymeric micelles was approximately four times higher than that of conventional oil-in-water micelles with Tween 60 (control), significantly inhibiting melanin production. These polymeric micelles hold significant promise as a transdermal drug delivery system for treating skin pigmentation.

Polymer micelles represent a novel delivery system that has emerged in recent years. For glabridin, polymer micelles enhance drug transdermal absorption and stability, making them suitable for intravenous injection and targeted drug delivery (183, 184). However, the preparation process is complex, with stringent requirements for storage and transportation conditions. Large-scale production requires further optimization (185). Currently, the regulatory pathway for polymer micelles remains unclear, and their large-scale clinical application continues to face challenges (186).

In summary, new drug delivery systems for glabridin, including liposomes, cyclodextrin complexes, polymer micelles, nanoparticles, nanoemulsions, nanosuspensions, and nanocomposites, have been extensively researched. These systems have made significant strides in improving the solubility, stability, and bioavailability of glabridin. Notably, glabridin is widely utilized in the cosmetics industry for its whitening effects. Current drug delivery systems for glabridin primarily focus on enhancing skin permeability and anti-melanin effects. However, there is a notable lack of research on its release rate, stability, accumulation, and targeting within the body. Furthermore, existing studies have mainly addressed issues related to drug distribution and release, with insufficient consideration of disease pathology and innovative treatment strategies. Future research should prioritize understanding disease pathogenesis and utilize functionalized materials sensitive to pH, redox conditions, temperature, light, sound, and magnetism. This approach aims to create synergistic effects between drugs and carriers, as well as between different drugs, while integrating diverse treatment methods to further explore the potential of glabridin in disease treatment. For detailed information, please refer to **Table 5**.

**Table 5:** Summary of the advantages and limitations of glabridin delivery systems.

Drug delivery systems		Advantage	Limitations	References
Liquid carrier system	Liposomes	Good biocompatibility, Enhanced bioavailability, Targeted delivery	Poor stability, Complex preparation process	(125, 126, 135, 139, 140, 142, 147, 148, 155, 157, 162, 164, 165, 168, 169, 178, 183, 184, 187)
	Nanosuspension	Suitable for insoluble drugs, Simple preparation process	The challenge of particle aggregation	
	Nanoemulsion	Enhanced bioavailability, Simple preparation process	High requirements for storage conditions, Poor stability	
Solid Carrier Systems	Nanoparticles	High drug-loading capacity, Targeted delivery, Good stability	Poor biocompatibility, High production costs	
	Nanocomplex	Diverse functionality, Smart drug release	Complex preparation process, High production costs	
	Metal-Organic frameworks	High specific surface area, Targeted delivery	Poor biocompatibility, Regulatory challenges	
Inclusion and polymer systems	Cyclodextrin inclusion complex	Enhance drug solubility, Good stability	Limited drug-loading capacity, Poor control over release rate	
	Polymeric micelles	High drug-loading capacity, Good biocompatibility, Good stability	Poor control over the release rate	

## 7 Conclusion and outlook

In recent years, plant extracts have been increasingly utilized in the clinical treatment of various diseases. Compared to synthetic drugs, plant extracts exhibit greater biodegradability, enhanced biocompatibility, and fewer side effects, making them considered safer alternatives (188). Malignant tumors pose a significant threat to human health and social development. Increasing evidence suggests that monomeric compounds derived from Chinese herbal medicine play a crucial role in anti-tumor therapy.

Glabridin demonstrates considerable promise in the realm of anti-tumor research. Its anti-tumor effects include inhibition of tumor cell proliferation, metastasis, and invasion; induction of apoptosis and cell cycle arrest; promotion of autophagy; suppression of angiogenesis; enhancement of chemotherapy sensitivity; and prevention of cancer. Compared to commonly used anti-tumor drugs, glabridin exhibits lower toxicity and side effects, along with good tolerance and safety. When combined with other anti-tumor drugs, glabridin can enhance therapeutic efficacy. Given its broad anti-tumor effects and its capacity to improve resistance to traditional chemotherapeutic drugs, glabridin can serve as an effective adjunct in small molecule targeted anti-tumor therapy, gene therapy, anti-angiogenesis therapy, and radiotherapy.

Although preclinical data indicate that glabridin has significant anti-cancer potential, significant challenges remain in translating preclinical findings to clinical applications. Current research on glabridin’s anti-tumor effects primarily focuses on *in vitro* cell studies. Future preclinical trials should include more animal models, particularly xenograft mouse models of various cancers, to evaluate glabridin’s anti-tumor efficacy and safety. Additionally, dose-optimization studies are necessary to establish the optimal dose range and assess long-term safety, especially regarding potential toxicity to target organs like the liver and kidneys. As a natural anti-tumor compound, glabridin holds promise for combination use with existing anti-cancer therapies, such as chemotherapy, radiotherapy, and immune checkpoint inhibitors. Future studies should explore combining it with standard anti-cancer drugs to evaluate the potential of combination therapy in overcoming drug resistance and enhancing efficacy.

Current drug delivery systems primarily focus on enhancing skin penetration and transdermal absorption, while research on dosage forms targeting tumors within the body remains limited. Future studies should aim to optimize drug delivery systems, particularly by enhancing the delivery efficiency, targeting, and therapeutic effects of glabridin through nano-carrier systems. For instance, polymeric nanoparticles (PNPs), lipid nanoparticles (LNPs), and nanogels can encapsulate glabridin effectively, enhancing its water solubility and controlling drug release. Adjusting particle size, surface modification, and drug loading can modify these nanocarriers to promote drug accumulation in tumor tissue, thereby enhancing therapeutic effects and minimizing side effects on normal tissues. Additionally, to further enhance glabridin’s therapeutic efficacy, developing tumor-targeted delivery systems is essential. Nanocarrier surfaces can be functionalized with specific antibodies, small-molecule peptides, or carbohydrate molecules to precisely target tumor cells. For example, glabridin can be combined with small peptides targeting EGFR or human epidermal growth factor receptor 2 (HER2) to achieve selective delivery to tumor sites.

In summary, glabridin exhibits substantial anti-tumor potential and holds considerable promise for future development and application. It is anticipated that further in-depth research will elucidate its potential and offer robust references and support for anti-tumor therapies.

## References

[1] Bray F Laversanne M Sung H Ferlay J Siegel RL Soerjomataram I. Global cancer statistics 2022: GLOBOCAN estimates of incidence and mortality worldwide for 36 cancers in 185 countries. CA Cancer J Clin. (2024) 74:229–63. doi: 10.3322/caac.21834 38572751

[2] Wu C Li M Meng H Liu Y Niu W Zhou Y. Analysis of status and countermeasures of cancer incidence and mortality in China. Sci China Life Sci. (2019) 62:640–7. doi: 10.1007/s11427-018-9461-5 30900169

[3] Huang Z Li G Lang Y An Q Chen H Zhang W. Research progress on the mechanisms of apoptosis induced by traditional Chinese medicine in lung cancer cells. Chin J Exp Tradit Med Formulae. (2021) 27:226–36. doi: 10.13422/j.cnki.syfjx.20212326

[4] Chen Y Fan W Zhao Y Liu M Hu L Zhang W. Progress in the regulation of immune cells in the tumor microenvironment by bioactive compounds of traditional chinese medicine. Molecules. (2024) 29:2374. doi: 10.3390/molecules29102374 38792234 PMC11124165

[5] Xie J Huang H Li X Ouyang L Wang L Liu D. The role of traditional chinese medicine in cancer immunotherapy: current status and future directions. Am J Chin Med. (2023) 51:1627–51. doi: 10.1142/S0192415X2350074X 37638827

[6] Xie Y Xu S Chen Z Song C Yan W. Unveiling the therapeutic potential of airpotato yam rhizome against colorectal cancer: a network pharmacology approach. Front Oncol. (2024) 14:1414766. doi: 10.3389/fonc.2024.1414766 39156706 PMC11327141

[7] Wang M Zhang F Zhou J Gong K Chen S Zhu X. Glabridin Ameliorates Alcohol-Caused Liver Damage by Reducing Oxidative Stress and Inflammation via p38 MAPK/Nrf2/NF-κB Pathway. Nutrients. (2023) 15:2157. doi: 10.3390/nu15092157 37432306 PMC10180694

[8] Muhammad H Salahuddin Z Akhtar T Aftab U Rafi A Hussain S. Immunomodulatory effect of glabridin in ovalbumin induced allergic asthma and its comparison with methylprednisolone in a preclinical rodent model. J Cell Biochem. (2023) 124:1503–15. doi: 10.1002/jcb.30459 37584465

[9] Long J Liu H Qiu Z Xiao Z Lu Z. Glabridin therapy reduces chronic allodynia, spinal microgliosis, and dendritic spine generation by inhibiting fractalkine-CX3CR1 signaling in a mouse model of tibial fractures. Brain Sci. (2023) 13:739. doi: 10.3390/brainsci13050739 37239211 PMC10216346

[10] Tan H Chen J Li Y Li Y Zhong Y Li G. Glabridin, a bioactive component of licorice, ameliorates diabetic nephropathy by regulating ferroptosis and the VEGF/Akt/ERK pathways. Mol Med. (2022) 28:58. doi: 10.1186/s10020-022-00481-w 35596156 PMC9123664

[11] Kaur K Narang RK Singh S. Glabridin mitigates TiO2NP induced cognitive deficit in adult zebrafish. Neurochem Int. (2023) 169:105585. doi: 10.1016/j.neuint.2023.105585 37499946

[12] Li S Wang Y Wu M Younis MH Olson AP Barnhart TE. Spleen-targeted glabridin-loaded nanoparticles regulate polarization of monocyte/macrophage (Mo/Mφ) for the treatment of cerebral ischemia-reperfusion injury. Adv Mater. (2022) 34:e2204976. doi: 10.1002/adma.202204976 35973230 PMC9594991

[13] Zhang J Wu X Zhong B Liao Q Wang X Xie Y. Review on the diverse biological effects of glabridin. Drug Des Devel Ther. (2023) 17:15–37. doi: 10.2147/DDDT.S385981 PMC9840373 36647530

[14] Bombelli A Araya-Cloutier C Vincken J-P Abee T den Besten HMW. Impact of food-relevant conditions and food matrix on the efficacy of prenylated isoflavonoids glabridin and 6,8-diprenylgenistein as potential natural preservatives against Listeria monocytogenes. Int J Food Microbiol. (2023) 390:110109. doi: 10.1016/j.ijfoodmicro.2023.110109 36806890

[15] Ciganović P Jakimiuk K Tomczyk M Zovko Končić M. Glycerolic licorice extracts as active cosmeceutical ingredients: extraction optimization, chemical characterization, and biological activity. Antioxidants (Basel). (2019) 8:445. doi: 10.3390/antiox8100445 31581512 PMC6826613

[16] Feng T Wei Y Lee RJ Zhao L. Liposomal curcumin and its application in cancer. Int J Nanomedicine. (2017) 12:6027–44. doi: 10.2147/IJN.S132434 PMC5573051 28860764

[17] Huang T Liu Y Zhang C. Pharmacokinetics and bioavailability enhancement of baicalin: A review. Eur J Drug Metab Pharmacokinet. (2019) 44:159–68. doi: 10.1007/s13318-018-0509-3 30209794

[18] Alizadeh SR Ebrahimzadeh MA. Quercetin derivatives: Drug design, development, and biological activities, a review. Eur J Med Chem. (2022) 229:114068. doi: 10.1016/j.ejmech.2021.114068 34971873

[19] Cao J Chen X Liang J Yu X-Q Xu A-L Chan E. Role of P-glycoprotein in the intestinal absorption of glabridin, an active flavonoid from the root of Glycyrrhiza glabra. Drug Metab Dispos. (2007) 35:539–53. doi: 10.1124/dmd.106.010801 17220245

[20] Ito C Oi N Hashimoto T Nakabayashi H Aoki F Tominaga Y. Absorption of dietary licorice isoflavan glabridin to blood circulation in rats. J Nutr Sci Vitaminol (Tokyo). (2007) 53:358–65. doi: 10.3177/jnsv.53.358 17934243

[21] Xie L Diao Z Xia J Zhang J Xu Y Wu Y. Comprehensive evaluation of metabolism and the contribution of the hepatic first-pass effect in the bioavailability of glabridin in rats. J Agric Food Chem. (2023) 71:1944–56. doi: 10.1021/acs.jafc.2c06460 36649475

[22] Simmler C Pauli GF Chen S-N. Phytochemistry and biological properties of glabridin. Fitoterapia. (2013) 90:160–84. doi: 10.1016/j.fitote.2013.07.003 PMC3795865 23850540

[23] Liang J Shang Y. Estrogen and cancer. Annu Rev Physiol. (2013) 75:225–40. doi: 10.1146/annurev-physiol-030212-183708 23043248

[24] Maximov PY Fan P Abderrahman B Curpan R Jordan VC. Estrogen receptor complex to trigger or delay estrogen-induced apoptosis in long-term estrogen deprived breast cancer. Front Endocrinol (Lausanne). (2022) 13:869562. doi: 10.3389/fendo.2022.869562 35360069 PMC8960923

[25] Bukato K Kostrzewa T Gammazza AM Gorska-Ponikowska M Sawicki S. Endogenous estrogen metabolites as oxidative stress mediators and endometrial cancer biomarkers. Cell Commun Signal. (2024) 22:205. doi: 10.1186/s12964-024-01583-0 38566107 PMC10985914

[26] Shin J Choi LS Jeon HJ Lee HM Kim SH Kim K-W. Synthetic glabridin derivatives inhibit LPS-induced inflammation via MAPKs and NF-κB pathways in RAW264.7 macrophages. Molecules. (2023) 28:2135. doi: 10.3390/molecules28052135 36903379 PMC10004008

[27] Feng Q Ye L Liu Y Gong Y Li H Wang Y. Determination of equilibrium solubility and oil-water partition coefficient of glabridin. J Tianjin Univ Tradit Chin Med. (2017) 36:54–6. doi: 10.11656/j.issn.1673-9043.2017.01.13

[28] Ao M Shi Y Cui Y Guo W Wang J Yu L. Factors influencing glabridin stability. Nat Prod Commun. (2010) 5:1907–12. doi: 10.1177/1934578X1000501214 21299118

[29] Aoki F Nakagawa K Kitano M Ikematsu H Nakamura K Yokota S. Clinical safety of licorice flavonoid oil (LFO) and pharmacokinetics of glabridin in healthy humans. J Am Coll Nutr. (2007) 26:209–18. doi: 10.1080/07315724.2007.10719603 17634165

[30] Liu S Lin H Chen Y Wang Y Zhang X Xiang Z. Metabolic profiling of glabridin in rat plasma, urine, bile, and feces after intragastric and intravenous administration. Eur J Drug Metab Pharmacokinet. (2022) 47:879–87. doi: 10.1007/s13318-022-00797-2 36107364

[31] Aoki F Honda S Kishida H Kitano M Arai N Tanaka H. Suppression by licorice flavonoids of abdominal fat accumulation and body weight gain in high-fat diet-induced obese C57BL/6J mice. Biosci Biotechnol Biochem. (2007) 71:206–14. doi: 10.1271/bbb.60463 17213668

[32] Nakagawa K Kitano M Kishida H Hidaka T Nabae K Kawabe M. 90-Day repeated-dose toxicity study of licorice flavonoid oil (LFO) in rats. Food Chem Toxicol. (2008) 46:2349–57. doi: 10.1016/j.fct.2008.03.015 18448224

[33] Nakagawa K Hidaka T Kitano M Asakura M Kamigaito T Noguchi T. Genotoxicity studies on licorice flavonoid oil (LFO). Food Chem Toxicol. (2008) 46:2525–32. doi: 10.1016/j.fct.2008.04.008 18502556

[34] Jiang F Li Y Mu J Hu C Zhou M Wang X. Glabridin inhibits cancer stem cell-like properties of human breast cancer cells: An epigenetic regulation of miR-148a/SMAd2 signaling. Mol Carcinog. (2016) 55:929–40. doi: 10.1002/mc.22333 25980823

[35] He B Li S Li Y Han J Li X Fang J. Effects of glabridin on the Malignant biological behavior of lung adenocarcinoma A549 cells and its molecular mechanisms. Chin J Cancer Biother. (2023) 30:672–80. doi: 10.3872/j.issn.1007-385x.2023.08.004

[36] Jamwal A Chand J Dash A Bhatt S Dhiman S Wazir P. Glabridin plays dual action to intensify anti-metastatic potential of paclitaxel via impeding CYP2C8 in liver and CYP2J2/EETs in tumor of an orthotopic mouse model of breast cancer. Chem Biol Interact. (2023) 382:110605. doi: 10.1016/j.cbi.2023.110605 37419298

[37] Gao C Wang Y Li D Tang X Zheng Q. Mechanistic study on glabridin-induced apoptosis in mouse melanoma B16F10 cells. Nat Prod Res Dev. (2017) 29:836–42. doi: 10.16333/j.1001-6880.2017.5.021

[38] Yang Z Bi Y Xu W Guo R Hao M Liang Y. Glabridin inhibits urothelial bladder carcinoma cell growth *in vitro* and *in vivo* by inducing cell apoptosis and cell cycle arrest. Chem Biol Drug Des. (2023) 101:581–92. doi: 10.1111/cbdd.14147 36098706

[39] Lin Y Xu J Lan H. Tumor-associated macrophages in tumor metastasis: biological roles and clinical therapeutic applications. J Hematol Oncol. (2019) 12:76. doi: 10.1186/s13045-019-0760-3 31300030 PMC6626377

[40] Castaneda M den Hollander P Kuburich NA Rosen JM Mani SA. Mechanisms of cancer metastasis. Semin Cancer Biol. (2022) 87:17–31. doi: 10.1016/j.semcancer.2022.10.006 36354098

[41] Budhu A Jia H-L Forgues M Liu C-G Goldstein D Lam A. Identification of metastasis-related microRNAs in hepatocellular carcinoma. Hepatology. (2008) 47:897–907. doi: 10.1002/hep.22160 18176954

[42] Pickup MW Owens P Moses HL. TGF-β, bone morphogenetic protein, and activin signaling and the tumor microenvironment. Cold Spring Harb Perspect Biol. (2017) 9:a022285. doi: 10.1101/cshperspect.a022285 28062564 PMC5411701

[43] Chandrasinghe P Cereser B Moorghen M Al Bakir I Tabassum N Hart A. Role of SMAD proteins in colitis-associated cancer: from known to the unknown. Oncogene. (2018) 37:1–7. doi: 10.1038/onc.2017.300 28869601

[44] Jiang F Mu J Wang X Ye X Si L Ning S. The repressive effect of miR-148a on TGF beta-SMADs signal pathway is involved in the glabridin-induced inhibition of the cancer stem cells-like properties in hepatocellular carcinoma cells. PloS One. (2014) 9:e96698. doi: 10.1371/journal.pone.0096698 24806207 PMC4013140

[45] Aprelikova O Palla J Hibler B Yu X Greer YE Yi M. Silencing of miR-148a in cancer-associated fibroblasts results in WNT10B-mediated stimulation of tumor cell motility. Oncogene. (2013) 32:3246–53. doi: 10.1038/onc.2012.351 PMC3711253 22890324

[46] Roy S Sunkara RR Parmar MY Shaikh S Waghmare SK. EMT imparts cancer stemness and plasticity: new perspectives and therapeutic potential. Front Biosci (Landmark Ed). (2021) 26:238–65. doi: 10.2741/4893 33049669

[47] Huang Y Hong W Wei X. The molecular mechanisms and therapeutic strategies of EMT in tumor progression and metastasis. J Hematol Oncol. (2022) 15:129. doi: 10.1186/s13045-022-01347-8 36076302 PMC9461252

[48] Loh C-Y Chai JY Tang TF Wong WF Sethi G Shanmugam MK. The E-cadherin and N-cadherin switch in epithelial-to-mesenchymal transition: signaling, therapeutic implications, and challenges. Cells. (2019) 8:1118. doi: 10.3390/cells8101118 31547193 PMC6830116

[49] Satelli A Li S. Vimentin in cancer and its potential as a molecular target for cancer therapy. Cell Mol Life Sci. (2011) 68:3033–46. doi: 10.1007/s00018-011-0735-1 PMC3162105 21637948

[50] Na T-Y Schecterson L Mendonsa AM Gumbiner BM. The functional activity of E-cadherin controls tumor cell metastasis at multiple steps. Proc Natl Acad Sci U.S.A. (2020) 117:5931–7. doi: 10.1073/pnas.1918167117 PMC7084067 32127478

[51] Gabarra-Niecko V Schaller MD Dunty JM. FAK regulates biological processes important for the pathogenesis of cancer. Cancer Metastasis Rev. (2003) 22:359–74. doi: 10.1023/a:1023725029589 12884911

[52] Hiscox S Nicholson RI. Src inhibitors in breast cancer therapy. Expert Opin Ther Targets. (2008) 12:757–67. doi: 10.1517/14728222.12.6.757 18479222

[53] Leung EL-H Tam IY-S Tin VP-C Chua DT-T Sihoe AD-L Cheng L-C. SRC promotes survival and invasion of lung cancers with epidermal growth factor receptor abnormalities and is a potential candidate for molecular-targeted therapy. Mol Cancer Res. (2009) 7:923–32. doi: 10.1158/1541-7786.MCR-09-0003 19491201

[54] Tsai Y-M Yang C-J Hsu Y-L Wu L-Y Tsai Y-C Hung J-Y. Glabridin inhibits migration, invasion, and angiogenesis of human non-small cell lung cancer A549 cells by inhibiting the FAK/rho signaling pathway. Integr Cancer Ther. (2011) 10:341–9. doi: 10.1177/1534735410384860 21059620

[55] Hsu Y-L Wu L-Y Hou M-F Tsai E-M Lee J-N Liang H-L. Glabridin, an isoflavan from licorice root, inhibits migration, invasion and angiogenesis of MDA-MB-231 human breast adenocarcinoma cells by inhibiting focal adhesion kinase/Rho signaling pathway. Mol Nutr Food Res. (2011) 55:318–27. doi: 10.1002/mnfr.201000148 20626003

[56] Mitra SK Schlaepfer DD. Integrin-regulated FAK-Src signaling in normal and cancer cells. Curr Opin Cell Biol. (2006) 18:516–23. doi: 10.1016/j.ceb.2006.08.011 16919435

[57] Niland S Riscanevo AX Eble JA. Matrix metalloproteinases shape the tumor microenvironment in cancer progression. Int J Mol Sci. (2021) 23:146. doi: 10.3390/ijms23010146 35008569 PMC8745566

[58] de Almeida LGN Thode H Eslambolchi Y Chopra S Young D Gill S. Matrix metalloproteinases: from molecular mechanisms to physiology, pathophysiology, and pharmacology. Pharmacol Rev. (2022) 74:712–68. doi: 10.1124/pharmrev.121.000349 35738680

[59] Jie Z Xie Z Zhao X Sun X Yu H Pan X. Glabridin inhibits osteosarcoma migration and invasion via blocking the p38- and JNK-mediated CREB-AP1 complexes formation. J Cell Physiol. (2019) 234:4167–78. doi: 10.1002/jcp.27171 30146723

[60] Liu L Chen J Zhang B Zheng Q. Comparative study on the anti-tumor metastasis effects of isoliquiritigenin and glabridin. Chin J Exp Tradit Med Formulae. (2013) 19:245–50. doi: 10.11653/syfj2013180245

[61] Hsieh M-J Lin C-W Yang S-F Chen M-K Chiou H-L. Glabridin inhibits migration and invasion by transcriptional inhibition of matrix metalloproteinase 9 through modulation of NF-κB and AP-1 activity in human liver cancer cells. Br J Pharmacol. (2014) 171:3037–50. doi: 10.1111/bph.12626 PMC4055204 24641665

[62] Elmore S. Apoptosis: a review of programmed cell death. Toxicol Pathol. (2007) 35:495–516. doi: 10.1080/01926230701320337 17562483 PMC2117903

[63] Pistritto G Trisciuoglio D Ceci C Garufi A D’Orazi G. Apoptosis as anticancer mechanism: function and dysfunction of its modulators and targeted therapeutic strategies. Aging (Albany NY). (2016) 8:603–19. doi: 10.18632/aging.100934 PMC4925817 27019364

[64] Hanahan D Weinberg RA. Hallmarks of cancer: the next generation. Cell. (2011) 144:646–74. doi: 10.1016/j.cell.2011.02.013 21376230

[65] Chelakkot C Chelakkot VS Shin Y Song K. Modulating glycolysis to improve cancer therapy. Int J Mol Sci. (2023) 24:2606. doi: 10.3390/ijms24032606 36768924 PMC9916680

[66] Akins NS Nielson TC Le HV. Inhibition of glycolysis and glutaminolysis: an emerging drug discovery approach to combat cancer. Curr Top Med Chem. (2018) 18:494–504. doi: 10.2174/1568026618666180523111351 29788892 PMC6110043

[67] Xu S Herschman HR. A tumor agnostic therapeutic strategy for hexokinase 1-null/hexokinase 2-positive cancers. Cancer Res. (2019) 79:5907–14. doi: 10.1158/0008-5472.CAN-19-1789 PMC12139393 31434645

[68] Forkasiewicz A Dorociak M Stach K Szelachowski P Tabola R Augoff K. The usefulness of lactate dehydrogenase measurements in current oncological practice. Cell Mol Biol Lett. (2020) 25:35. doi: 10.1186/s11658-020-00228-7 32528540 PMC7285607

[69] Sharma D Singh M Rani R. Role of LDH in tumor glycolysis: Regulation of LDHA by small molecules for cancer therapeutics. Semin Cancer Biol. (2022) 87:184–95. doi: 10.1016/j.semcancer.2022.11.007 36371026

[70] Ancey P-B Contat C Meylan E. Glucose transporters in cancer - from tumor cells to the tumor microenvironment. FEBS J. (2018) 285:2926–43. doi: 10.1111/febs.14577 29893496

[71] Li L Li G Xie Y. Regulatory effects of glabridin and quercetin on energy metabolism in breast cancer cells. Chin J Chin Mater Med. (2019) 44:3786–91. doi: 10.19540/j.cnki.cjcmm.20190505.401 31602954

[72] Yang Y Karakhanova S Hartwig W D’Haese JG Philippov PP Werner J. Mitochondria and mitochondrial ROS in cancer: novel targets for anticancer therapy. J Cell Physiol. (2016) 231:2570–81. doi: 10.1002/jcp.25349 26895995

[73] Cui X Cui M. Glabridin induces paraptosis-like cell death via ER stress in breast cancer cells. Heliyon. (2022) 8:e10607. doi: 10.1016/j.heliyon.2022.e10607 36158101 PMC9489725

[74] Talukdar S Emdad L Das SK Fisher PB. EGFR: An essential receptor tyrosine kinase-regulator of cancer stem cells. Adv Cancer Res. (2020) 147:161–88. doi: 10.1016/bs.acr.2020.04.003 32593400

[75] Li X Zhao L Chen C Nie J Jiao B. Can EGFR be a therapeutic target in breast cancer? Biochim Biophys Acta Rev Cancer. (2022) 1877:188789. doi: 10.1016/j.bbcan.2022.188789 36064121

[76] Ghosh A Ghosh D Mukerjee N Maitra S Das P Dey A. The efficient activity of glabridin and its derivatives against EGFRmediated inhibition of breast cancer. Curr Med Chem. (2024) 31:573–94. doi: 10.2174/0929867330666230303120942 36872353

[77] Zhu K Li K Wang H Kang L Dang C Zhang Y. Discovery of glabridin as potent inhibitor of epidermal growth factor receptor in SK-BR-3 cell. Pharmacology. (2019) 104:113–25. doi: 10.1159/000496798 31167205

[78] Cardano M Tribioli C Prosperi E. Targeting proliferating cell nuclear antigen (PCNA) as an effective strategy to inhibit tumor cell proliferation. Curr Cancer Drug Targets. (2020) 20:240–52. doi: 10.2174/1568009620666200115162814 31951183

[79] He Y Sun MM Zhang GG Yang J Chen KS Xu WW. Targeting PI3K/Akt signal transduction for cancer therapy. Signal Transduct Target Ther. (2021) 6:425. doi: 10.1038/s41392-021-00828-5 34916492 PMC8677728

[80] Tan W Zhou S Kang H Jiang X Mao Z Yang K. Mechanism of glabridin in the treatment of castration-resistant prostate cancer based on network pharmacology and experimental validation. Nat Prod Res Dev. (2023) 35:310–318, 259. doi: 10.16333/j.1001-6880.2023.2.015

[81] Zhang F Wang F Li W Liang L Sang X. The toxicity mechanism of glabridin in prostate cancer cells is involved in reactive oxygen species-dependent PI3K/Akt pathway: Integrated utilization of bioinformatic analysis and *in vitro* test validation. Environ Toxicol. (2022) 37:2937–46. doi: 10.1002/tox.23649 36029289

[82] Moloney JN Cotter TG. ROS signalling in the biology of cancer. Semin Cell Dev Biol. (2018) 80:50–64. doi: 10.1016/j.semcdb.2017.05.023 28587975

[83] Hua H Kong Q Zhang H Wang J Luo T Jiang Y. Targeting mTOR for cancer therapy. J Hematol Oncol. (2019) 12:71. doi: 10.1186/s13045-019-0754-1 31277692 PMC6612215

[84] Li T Li H Xia W Li M Niu Y Fu X. Investigation of the effects of glabridin on the proliferation, apoptosis, and migration of the human colon cancer cell lines SW480 and SW620 and its mechanism based on reverse virtual screening and proteomics. Oxid Med Cell Longev. (2023) 2023:1117431. doi: 10.1155/2023/1117431 36644579 PMC9836797

[85] Abdelrahman KS Hassan HA Abdel-Aziz SA Marzouk AA Narumi A Konno H. JNK signaling as a target for anticancer therapy. Pharmacol Rep. (2021) 73:405–34. doi: 10.1007/s43440-021-00238-y 33710509

[86] Wu Q Wu W Jacevic V Franca TCC Wang X Kuca K. Selective inhibitors for JNK signalling: a potential targeted therapy in cancer. J Enzyme Inhib Med Chem. (2020) 35:574–83. doi: 10.1080/14756366.2020.1720013 PMC7034130 31994958

[87] Zou X Blank M. Targeting p38 MAP kinase signaling in cancer through post-translational modifications. Cancer Lett. (2017) 384:19–26. doi: 10.1016/j.canlet.2016.10.008 27725227

[88] Yang X Zou D Ding Y Zuo L Zhou Q Wang Y. Effects of glabridin on proliferation and apoptosis of colorectal cancer RKO cells. J Anhui Med Univ. (2017) 52:42–6. doi: 10.19405/j.cnki.issn1000-1492.2017.01.009

[89] Huang H-L Hsieh M-J Chien M-H Chen H-Y Yang S-F Hsiao P-C. Glabridin mediate caspases activation and induces apoptosis through JNK1/2 and p38 MAPK pathway in human promyelocytic leukemia cells. PloS One. (2014) 9:e98943. doi: 10.1371/journal.pone.0098943 24901249 PMC4047044

[90] Liu J Peng Y Wei W. Cell cycle on the crossroad of tumorigenesis and cancer therapy. Trends Cell Biol. (2022) 32:30–44. doi: 10.1016/j.tcb.2021.07.001 34304958 PMC8688170

[91] Tchakarska G Sola B. The double dealing of cyclin D1. Cell Cycle. (2020) 19:163–78. doi: 10.1080/15384101.2019.1706903 PMC6961668 31885322

[92] Wang Z Luo S Wan Z Chen C Zhang X Li B. Glabridin arrests cell cycle and inhibits proliferation of hepatocellular carcinoma by suppressing braf/MEK signaling pathway. Tumour Biol. (2016) 37:5837–46. doi: 10.1007/s13277-015-4177-5 26586395

[93] Jin T George Fantus I Sun J. Wnt and beyond Wnt: multiple mechanisms control the transcriptional property of beta-catenin. Cell Signal. (2008) 20:1697–704. doi: 10.1016/j.cellsig.2008.04.014 18555664

[94] Yu F Yu C Li F Zuo Y Wang Y Yao L. Wnt/β-catenin signaling in cancers and targeted therapies. Signal Transduct Target Ther. (2021) 6:307. doi: 10.1038/s41392-021-00701-5 34456337 PMC8403677

[95] Zhang Y Wang X. Targeting the Wnt/β-catenin signaling pathway in cancer. J Hematol Oncol. (2020) 13:165. doi: 10.1186/s13045-020-00990-3 33276800 PMC7716495

[96] Huang S Li C Huang J Mo D Luo P Wang H. Effects and mechanisms of glabridin on proliferation, invasion, and apoptosis of cervical cancer HeLa cells. Mod Oncol. (2020) 28:3289–93. doi: 10.3969/j.issn.1672-4992.2020.19.003

[97] Levy JMM Towers CG Thorburn A. Targeting autophagy in cancer. Nat Rev Cancer. (2017) 17:528–42. doi: 10.1038/nrc.2017.53 PMC5975367 28751651

[98] Tanida I Ueno T Kominami E. LC3 conjugation system in mammalian autophagy. Int J Biochem Cell Biol. (2004) 36:2503–18. doi: 10.1016/j.biocel.2004.05.009 PMC7129593 15325588

[99] Klicka K Grzywa TM Mielniczuk A Klinke A Włodarski PK. The role of miR-200 family in the regulation of hallmarks of cancer. Front Oncol. (2022) 12:965231. doi: 10.3389/fonc.2022.965231 36158660 PMC9492973

[100] Hsieh M-J Chen M-K Chen C-J Hsieh M-C Lo Y-S Chuang Y-C. Glabridin induces apoptosis and autophagy through JNK1/2 pathway in human hepatoma cells. Phytomedicine. (2016) 23:359–66. doi: 10.1016/j.phymed.2016.01.005 27002406

[101] Lugano R Ramachandran M Dimberg A. Tumor angiogenesis: causes, consequences, challenges and opportunities. Cell Mol Life Sci. (2020) 77:1745–70. doi: 10.1007/s00018-019-03351-7 PMC7190605 31690961

[102] Liu Z-L Chen H-H Zheng L-L Sun L-P Shi L. Angiogenic signaling pathways and anti-angiogenic therapy for cancer. Signal Transduct Target Ther. (2023) 8:198. doi: 10.1038/s41392-023-01460-1 37169756 PMC10175505

[103] Mu J Zhu D Shen Z Ning S Liu Y Chen J. The repressive effect of miR-148a on Wnt/β-catenin signaling involved in Glabridin-induced anti-angiogenesis in human breast cancer cells. BMC Cancer. (2017) 17:307. doi: 10.1186/s12885-017-3298-1 28464803 PMC5414299

[104] Zraik IM Heß-Busch Y. Management of chemotherapy side effects and their long-term sequelae. Urologe A. (2021) 60:862–71. doi: 10.1007/s00120-021-01569-7 34185118

[105] Pomeroy AE Schmidt EV Sorger PK Palmer AC. Drug independence and the curability of cancer by combination chemotherapy. Trends Cancer. (2022) 8:915–29. doi: 10.1016/j.trecan.2022.06.009 PMC9588605 35842290

[106] Wei Y Yang P Cao S Zhao L. The combination of curcumin and 5-fluorouracil in cancer therapy. Arch Pharm Res. (2018) 41:1–13. doi: 10.1007/s12272-017-0979-x 29230689

[107] Valencia-Lazcano AA Hassan D Pourmadadi M Shamsabadipour A Behzadmehr R Rahdar A. 5-Fluorouracil nano-delivery systems as a cutting-edge for cancer therapy. Eur J Med Chem. (2023) 246:114995. doi: 10.1016/j.ejmech.2022.114995 36493619

[108] Zhang L Chen H Wang M Song X Ding F Zhu J. Effects of glabridin combined with 5-fluorouracil on the proliferation and apoptosis of gastric cancer cells. Oncol Lett. (2018) 15:7037–45. doi: 10.3892/ol.2018.8260 PMC5920351 29725429

[109] Legha SS. Tamoxifen in the treatment of breast cancer. Ann Intern Med. (1988) 109:219–28. doi: 10.7326/0003-4819-109-3-219 3291659

[110] Gallego-Jara J Lozano-Terol G Sola-Martínez RA Cánovas-Díaz M de Diego Puente T. A compressive review about taxol^®^: history and future challenges. Molecules. (2020) 25:5986. doi: 10.3390/molecules25245986 33348838 PMC7767101

[111] Škubník J Svobodová Pavlíčková V Ruml T Rimpelová S. Autophagy in cancer resistance to paclitaxel: Development of combination strategies. BioMed Pharmacother. (2023) 161:114458. doi: 10.1016/j.biopha.2023.114458 36889112

[112] Chen C Lu L Yan S Yi H Yao H Wu D. Autophagy and doxorubicin resistance in cancer. Anticancer Drugs. (2018) 29:1–9. doi: 10.1097/CAD.0000000000000572 29099416

[113] Lin H Ai D Liu Q Wang X Chen Q Hong Z. Natural isoflavone glabridin targets PI3Kγ as an adjuvant to increase the sensitivity of MDA-MB-231 to tamoxifen and DU145 to paclitaxel. J Steroid Biochem Mol Biol. (2024) 236:106426. doi: 10.1016/j.jsbmb.2023.106426 37984749

[114] Carvalho C Santos RX Cardoso S Correia S Oliveira PJ Santos MS. Doxorubicin: the good, the bad and the ugly effect. Curr Med Chem. (2009) 16:3267–85. doi: 10.2174/092986709788803312 19548866

[115] Qian J Xia M Liu W Li L Yang J Mei Y. Glabridin resensitizes p-glycoprotein-overexpressing multidrug-resistant cancer cells to conventional chemotherapeutic agents. Eur J Pharmacol. (2019) 852:231–43. doi: 10.1016/j.ejphar.2019.04.002 30959046

[116] Modarresi M Hajialyani M Moasefi N Ahmadi F Hosseinzadeh L. Evaluation of the cytotoxic and apoptogenic effects of glabridin and its effect on cytotoxicity and apoptosis induced by doxorubicin toward cancerous cells. Adv Pharm Bull. (2019) 19:481–9. doi: 10.15171/apb.2019.057 PMC6773930 31592119

[117] Huang K Liu Y Tang H Qiu M Li C Duan C. Glabridin prevents doxorubicin-induced cardiotoxicity through gut microbiota modulation and colonic macrophage polarization in mice. Front Pharmacol. (2019) 10:107. doi: 10.3389/fphar.2019.00107 30833897 PMC6387923

[118] Van Laethem A Garmyn M Agostinis P. Starting and propagating apoptotic signals in UVB irradiated keratinocytes. Photochem Photobiol Sci. (2009) 8:299–308. doi: 10.1039/b813346h 19255669

[119] Peng G Li Y Zeng Y Sun B Zhang L Liu Q. Effect of glabridin combined with bakuchiol on UVB-induced skin damage and its underlying mechanism: An experimental study. J Cosmet Dermatol. (2024) 23:2256–69. doi: 10.1111/jocd.16259 38497297

[120] Veratti E Rossi T Giudice S Benassi L Bertazzoni G Morini D. 18beta-glycyrrhetinic acid and glabridin prevent oxidative DNA fragmentation in UVB-irradiated human keratinocyte cultures. Anticancer Res. (2011) 31:2209–15. 21737643

[121] Su Wei Poh M Voon Chen Yong P Viseswaran N Chia YY. Estrogenicity of glabridin in Ishikawa cells. PloS One. (2015) 10:e0121382. doi: 10.1371/journal.pone.0121382 25816349 PMC4376725

[122] Jen SH Wei MPS Yin ACY. The combinatory effects of glabridin and tamoxifen on ishikawa and MCF-7 cell lines. Nat Prod Commun. (2015) 10:1573–6. doi: 10.1177/1934578X1501000922 26594762

[123] Hassan S Prakash G Ozturk A Saghazadeh S Sohail MF Seo J. Evolution and clinical translation of drug delivery nanomaterials. Nano Today. (2017) 15:91–106. doi: 10.1016/j.nantod.2017.06.008 29225665 PMC5720147

[124] Guimarães D Cavaco-Paulo A Nogueira E. Design of liposomes as drug delivery system for therapeutic applications. Int J Pharm. (2021) 601:120571. doi: 10.1016/j.ijpharm.2021.120571 33812967

[125] Large DE Abdelmessih RG Fink EA Auguste DT. Liposome composition in drug delivery design, synthesis, characterization, and clinical application. Adv Drug Delivery Rev. (2021) 176:113851. doi: 10.1016/j.addr.2021.113851 34224787

[126] Zununi Vahed S Salehi R Davaran S Sharifi S. Liposome-based drug co-delivery systems in cancer cells. Mater Sci Eng C Mater Biol Appl. (2017) 71:1327–41. doi: 10.1016/j.msec.2016.11.073 27987688

[127] Huang J He X. Preparation and quality evaluation of glabridin liposomes. Biomass Chem Eng. (2016) 50:8–12. doi: 10.3969/j.issn.1673-5854.2016.03.002

[128] Zhang C Lu Y Ai Y Xu X Zhu S Zhang B. Glabridin liposome ameliorating UVB-induced erythema and lethery skin by suppressing inflammatory cytokine production. J Microbiol Biotechnol. (2021) 31:630–6. doi: 10.4014/jmb.2011.11006 PMC9706034 33526759

[129] Zhang C Luo S Zhang Z Niu Y Zhang W. Evaluation of Glabridin loaded nanostructure lipid carriers. J Taiwan Inst Chem Eng. (2017) 71:338–43. doi: 10.1016/j.jtice.2016.11.010

[130] Wang Y Wu K Li S Li X He Y. *In vivo* confocal Raman spectroscopy investigation of glabridin liposomes dermal penetration process in human skin. Vib Spectrosc. (2023) 129:103610. doi: 10.1016/j.vibspec.2023.103610

[131] Kalave S Chatterjee B Shah P Misra A. Transdermal delivery of macromolecules using nano lipid carriers. Curr Pharm Des. (2021) 27:4330–40. doi: 10.2174/1381612827666210820095330 34414868

[132] kbarzadeh A Rezaei-Sadabady R Davaran S Joo SW Zarghami N Hanifehpour Y. Liposome: classification, preparation, and applications. Nanoscale Res Lett. (2013) 8:102. doi: 10.1186/1556-276X-8-102 23432972 PMC3599573

[133] Liu P Chen G Zhang J. A review of liposomes as a drug delivery system: current status of approved products, regulatory environments, and future perspectives. Molecules. (2022) 27:1372. doi: 10.3390/molecules27041372 35209162 PMC8879473

[134] Thotakura N Gupta MM Rajawat JS Raza K. Promises of lipid-based drug delivery systems in the management of breast cancer. Curr Pharm design. (2021) 27: 4568–77. doi: 10.2174/1381612827666210728104318 34323182

[135] Elgart A Cherniakov I Aldouby Y Domb AJ Hoffman A. Lipospheres and pro-nano lipospheres for delivery of poorly water soluble compounds. Chem Phys Lipids. (2012) 165:438–53. doi: 10.1016/j.chemphyslip.2012.01.007 22342324

[136] Aldeeb MME Wilar G Suhandi C Elamin KM Wathoni N. Nanosuspension-based drug delivery systems for topical applications. Int J Nanomedicine. (2024) 19:825–44. doi: 10.2147/IJN.S447429 PMC10824615 38293608

[137] Jacob S Nair AB Shah J. Emerging role of nanosuspensions in drug delivery systems. Biomater Res. (2020) 24:3. doi: 10.1186/s40824-020-0184-8 31969986 PMC6964012

[138] Wang WP Hul J Sui H Zhao YS Feng J Liu C. Glabridin nanosuspension for enhanced skin penetration: formulation optimization, *in vitro* and *in vivo* evaluation. Pharmazie. (2016) 71:252–7. 27348968

[139] Bujňáková Z Dutková E Baláž M Turianicová E Baláž P. Stability studies of As4S4 nanosuspension prepared by wet milling in Poloxamer 407. Int J Pharm. (2015) 478:187–92. doi: 10.1016/j.ijpharm.2014.11.043 25448581

[140] Ahire E Thakkar S Darshanwad M Misra M. Parenteral nanosuspensions: a brief review from solubility enhancement to more novel and specific applications. Acta Pharm Sin B. (2018) 8:733–55. doi: 10.1016/j.apsb.2018.07.011 PMC6146387 30245962

[141] Roy A Nishchaya K Rai VK. Nanoemulsion-based dosage forms for the transdermal drug delivery applications: A review of recent advances. Expert Opin Drug delivery. (2022) 19:303–19. doi: 10.1080/17425247.2022.2045944 35196938

[142] Jethara SI Patel AD Patel MR Patel MS Patel KR. Recent survey on nanosuspension: a patent overview. Recent Pat Drug Delivery Formul. (2015) 9:65–78. doi: 10.2174/1872211308666141028214003 25354346

[143] Anton N Vandamme TF. Nano-emulsions and micro-emulsions: clarifications of the critical differences. Pharm Res. (2011) 28:978–85. doi: 10.1007/s11095-010-0309-1 21057856

[144] Yang X Zhao K Jiang J. Application of nanoemulsion drug delivery systems. Guangdong Agric Sci. (2011) 38:125–7. doi: 10.16768/j.issn.1004-874x.2011.07.073

[145] Liu C Hu J Sui H Zhao Q Zhang X Wang W. Enhanced skin permeation of glabridin using eutectic mixture-based nanoemulsion. Drug Delivery Transl Res. (2017) 7:325–32. doi: 10.1007/s13346-017-0359-6 28188607

[146] Hörmann K Zimmer A. Drug delivery and drug targeting with parenteral lipid nanoemulsions - A review. J Control Release. (2016) 223:85–98. doi: 10.1016/j.jconrel.2015.12.016 26699427

[147] Mushtaq A Mohd Wani S Malik AR Gull A Ramniwas S Ahmad Nayik G. Recent insights into Nanoemulsions: Their preparation, properties and applications. Food Chem X. (2023) 18:100684. doi: 10.1016/j.fochx.2023.100684 37131847 PMC10149285

[148] Singh Y Meher JG Raval K Khan FA Chaurasia M Jain NK. Nanoemulsion: Concepts, development and applications in drug delivery. J Control Release. (2017) 252:28–49. doi: 10.1016/j.jconrel.2017.03.008 28279798

[149] Ferreira Soares DC Domingues SC Viana DB Tebaldi ML. Polymer-hybrid nanoparticles: Current advances in biomedical applications. BioMed Pharmacother. (2020) 131:110695. doi: 10.1016/j.biopha.2020.110695 32920512

[150] Guo Y Liu H Li S He G Chen Q Mei Q. Research progress on stimulus-responsive and targeted mesoporous silica nanoparticles for antitumor drug delivery. Chem Biochem Eng. (2021) 38:9–16. doi: 10.3969/j.issn.1672-5425.2021.08.002

[151] Chen Y Ping Q. Research progress on oral drug-loaded nanoparticles. Prog Pharm Sci. (2004) 10:451–5. doi: 10.3969/j.issn.1001-5094.2004.10.005

[152] Chen H Zhuang J Wu X Shen X Zhang Q Zhang W. Preparation of the chitosan/poly-γ-glutamic acid/glabrid in hybrid nanoparticles and study on its releasing property. Curr Drug Delivery. (2023) 20:1195–205. doi: 10.2174/1567201819666220513122319 35570557

[153] Liu Y Yang G Jin S Xu L Zhao C-X. Development of high-drug-loading nanoparticles. Chempluschem. (2020) 85:2143–57. doi: 10.1002/cplu.202000496 32864902

[154] Shi J Kantoff PW Wooster R Farokhzad OC. Cancer nanomedicine: progress, challenges and opportunities. Nat Rev Cancer. (2017) 17:20–37. doi: 10.1038/nrc.2016.108 27834398 PMC5575742

[155] Elespuru R Pfuhler S Aardema MJ Chen T Doak SH Doherty A. Genotoxicity assessment of nanomaterials: recommendations on best practices, assays, and methods. Toxicol Sci. (2018) 164:391–416. doi: 10.1093/toxsci/kfy100 29701824

[156] D’Angelo NA Noronha MA Kurnik IS Câmara MCC Vieira JM Abrunhosa L. Curcumin encapsulation in nanostructures for cancer therapy: A 10-year overview. Int J Pharm. (2021) 604:120534. doi: 10.1016/j.ijpharm.2021.120534 33781887

[157] Sruthi P Madhava Naidu M Rao PJ. Valorization of cashew nut testa phenolics through nano-complexes stabilized with whey protein isolate and β-cyclodextrin: Characterization, anti-oxidant activity, stability and *in vitro* release. Food Res Int. (2024) 181:114110. doi: 10.1016/j.foodres.2024.114110 38448109

[158] Jafernik K Ładniak A Blicharska E Czarnek K Ekiert H Wiącek AE. Chitosan-based nanoparticles as effective drug delivery systems-A review. Molecules. (2023) 28:1963. doi: 10.3390/molecules28041963 36838951 PMC9959713

[159] Park Y-S Park H-J Lee J. Stabilization of glabridin by chitosan nano-complex. J Korean Soc Appl Biol Chem. (2012) 55:457–62. doi: 10.1007/s13765-012-2001-0

[160] Liu C Liu Z Sun X Zhang S Wang S Feng F. Fabrication and characterization of β-lactoglobulin-based nanocomplexes composed of chitosan oligosaccharides as vehicles for delivery of astaxanthin. J Agric Food Chem. (2018) 66:6717–26. doi: 10.1021/acs.jafc.8b00834 29883537

[161] Wei Y Vriesekoop F Yuan Q Liang H. [amp]]beta;-lactoglobulin as a nanotransporter for glabridin: exploring the binding properties and bioactivity influences. ACS Omega. (2018) 3:12246–52. doi: 10.1021/acsomega.8b01576 PMC6645583 31459299

[162] Cho K Wang X Nie S Chen ZG Shin DM. Therapeutic nanoparticles for drug delivery in cancer. Clin Cancer Res. (2008) 14:1310–6. doi: 10.1158/1078-0432.CCR-07-1441 18316549

[163] Kang M Lee SH Kwon M Byun J Kim D Kim C. Nanocomplex-mediated *in vivo* programming to chimeric antigen receptor-M1 macrophages for cancer therapy. Adv Mater. (2021) 33:e2103258. doi: 10.1002/adma.202103258 34510559

[164] Xu Z Zhou Z Yang X Thakur A Han N Li H-T. Determining M2 macrophages content for the anti-tumor effects of metal-organic framework-encapsulated pazopanib nanoparticles in breast cancer. J Nanobiotechnology. (2024) 22:429. doi: 10.1186/s12951-024-02694-z 39033109 PMC11264935

[165] Chiñas-Rojas LE Domínguez JE Herrera LÁA González-Jiménez FE Colorado-Peralta R Arenzano Altaif JA. Exploring synthesis strategies and interactions between MOFs and drugs for controlled drug loading and release, characterizing interactions through advanced techniques. ChemMedChem. (2024) 19:e202400144. doi: 10.1002/cmdc.202400144 39049537

[166] Ke Q Jiang K Li H Zhang L Chen B. Hierarchically micro-, meso-, and macro-porous MOF nanosystems for localized cross-scale dual-biomolecule loading and guest-carrier cooperative anticancer therapy. ACS Nano. (2024) 18:21911–24. doi: 10.1021/acsnano.4c02288 39102565

[167] Chen L Liu Z Zhao X Liu L Xin X Liang H. Self-assembled pH-responsive metal-organic frameworks for enhancing the encapsulation and anti-oxidation and melanogenesis inhibition activities of glabridin. Molecules. (2022) 27:3908. doi: 10.3390/molecules27123908 35745031 PMC9227565

[168] Mhettar P Kale N Pantwalawalkar J Nangare S Jadhav N. Metal-organic frameworks: Drug delivery applications and future prospects. ADMET DMPK. (2024) 12:27–62. doi: 10.5599/admet.2057 38560715 PMC10974818

[169] Nguyen NTT Nguyen TTT Ge S Liew RK Nguyen DTC Tran TV. Recent progress and challenges of MOF-based nanocomposites in bioimaging, biosensing and biocarriers for drug delivery. Nanoscale Adv. (2024) 6:1800–21. doi: 10.1039/d3na01075a PMC10964756 38545292

[170] Lu Q Sun L. Research progress on new dosage forms of baicalein preparations. J Shenyang Pharm Univ. (2023) 40:1253–64. doi: 10.14066/j.cnki.cn21-1349/r.2022.0060

[171] van der Veen BA van Alebeek GJ Uitdehaag JC Dijkstra BW Dijkhuizen L. The three transglycosylation reactions catalyzed by cyclodextrin glycosyltransferase from Bacillus circulans (strain 251) proceed via different kinetic mechanisms. Eur J Biochem. (2000) 267:658–65. doi: 10.1046/j.1432-1327.2000.01031.x 10651801

[172] Kamaraj M Suresh Babu P Shyamalagowri S Pavithra MKS Aravind J Kim W. β-cyclodextrin polymer composites for the removal of pharmaceutical substances, endocrine disruptor chemicals, and dyes from aqueous solution- A review of recent trends. J Environ Manage. (2024) 351:119830. doi: 10.1016/j.jenvman.2023.119830 38141340

[173] Wei Y Zhang J Zhou Y Bei W Li Y Yuan Q. Characterization of glabridin/hydroxypropyl-β-cyclodextrin inclusion complex with robust solubility and enhanced bioactivity. Carbohydr Polym. (2017) 159:152–60. doi: 10.1016/j.carbpol.2016.11.093 28038744

[174] Li D Fan J Yao P Ren G Du L Zhang X. Release characteristics, mucosal permeability, and cellular uptake of glabridin/hydroxypropyl-β-cyclodextrin inclusion complex. Food Sci. (2023) 44:16–25. doi: 10.7506/spkx1002-6630-20230120-155

[175] Wang W Tang K Huang C Liu A. Fabrication of sulfobutylether-β-cyclodextrin/glabridin inclusion complex for promoting bioactivities. Arch Pharm (Weinheim). (2024) 357:e2400082. doi: 10.1002/ardp.202400082 38724255

[176] Yao P Fan J Li D Zhang X Ren G Du L. Preparation and properties of glabridin/cyclodextrin solid inclusion complex. Food Sci. (2022) 43:9–18. doi: 10.7506/spkx1002-6630-20210901-006

[177] Jansook P Ogawa N Loftsson T. Cyclodextrins: structure, physicochemical properties and pharmaceutical applications. Int J Pharm. (2018) 535:272–84. doi: 10.1016/j.ijpharm.2017.11.018 29138045

[178] Benkő B-M Tóth G Moldvai D Kádár S Szabó E Szabó Z-I. Cyclodextrin encapsulation enabling the anticancer repositioning of disulfiram: Preparation, analytical and *in vitro* biological characterization of the inclusion complexes. Int J Pharm. (2024) 657:124187. doi: 10.1016/j.ijpharm.2024.124187 38697585

[179] Chaudhari P Ghate VM Lewis SA. Supramolecular cyclodextrin complex: Diversity, safety, and applications in ocular therapeutics. Exp Eye Res. (2019) 189:107829. doi: 10.1016/j.exer.2019.107829 31605685

[180] Serrano-Martínez A Victoria-Montesinos D García-Muñoz AM Hernández-Sánchez P Lucas-Abellán C González-Louzao R. A systematic review of clinical trials on the efficacy and safety of CRLX101 cyclodextrin-based nanomedicine for cancer treatment. Pharmaceutics. (2023) 15:1824. doi: 10.3390/pharmaceutics15071824 37514011 PMC10383811

[181] Manjappa AS Kumbhar PS Disouza JI Patravale VB. Polymeric mixed micelles: improving the anticancer efficacy of single-copolymer micelles. Crit Rev Ther Drug Carrier Syst. (2019) 36:1–58. doi: 10.1615/CritRevTherDrugCarrierSyst.2018020481 30789817

[182] Seino H Arai Y Nagao N Ozawa N Hamada K. Efficient percutaneous delivery of the antimelanogenic agent glabridin using cationic amphiphilic chitosan micelles. PloS One. (2016) 11:e0164061. doi: 10.1371/journal.pone.0164061 27695112 PMC5047624

[183] Tanbour R Martins AM Pitt WG Husseini GA. Drug delivery systems based on polymeric micelles and ultrasound: A review. Curr Pharm Des. (2016) 22:2796–807. doi: 10.2174/1381612822666160217125215 26898742

[184] Shaikh R Bhattacharya S. Polymeric micelles in colorectal cancer therapy: A comprehensive review of nano-drug delivery strategies, copolymer types, physicochemical characteristics, and therapeutic applications. Curr Med Chem. (2024). doi: 10.2174/0109298673306752240726104241 39092735

[185] Yang Y Xu S Chen B Tian Y Zou S Chen B. Preparation of amphiphilic polymeric nanomicelles and research progress on their drug-loading methods. Chem Adhes. (2024) 46:397–401. doi: 10.3969/j.issn.1001-0017.2024.04.018

[186] Junnuthula V Kolimi P Nyavanandi D Sampathi S Vora LK Dyawanapelly S. Polymeric micelles for breast cancer therapy: recent updates, clinical translation and regulatory considerations. Pharmaceutics. (2022) 14:1860. doi: 10.3390/pharmaceutics14091860 36145608 PMC9501124

[187] Sarabia-Vallejo Á Caja MDM Olives AI Martín MA Menéndez JC. Cyclodextrin inclusion complexes for improved drug bioavailability and activity: synthetic and analytical aspects. Pharmaceutics. (2023) 15:2345. doi: 10.3390/pharmaceutics15092345 37765313 PMC10534465

[188] He J Zhang H-P. Research progress on the anti-tumor effect of Naringin. Front Pharmacol. (2023) 14:1217001. doi: 10.3389/fphar.2023.1217001 37663256 PMC10469811

